# Computational prediction for the formation of amides and thioamides in the gas phase interstellar medium

**DOI:** 10.3389/fchem.2025.1615586

**Published:** 2025-06-30

**Authors:** Mohamad Akbar Ali, Sorakayala Thripati

**Affiliations:** ^1^ Department of Chemistry, Khalifa University of Science and Technology, Abu Dhabi, United Arab Emirates; ^2^ Center for Catalysis and Separations, Khalifa University of Science and Technology, Abu Dhabi, United Arab Emirates

**Keywords:** astrochemistry, interstellar chemical reactions, reactive intermediates, amino acids, peptide bonds, thioamides, *ab initio*

## Abstract

Amino acids and amide bonds (−C(O)−NH−) are the essential components of proteins, which serve as the foundation of life on Earth. As a result, molecules containing peptide bonds are of great interest in studies related to the origin of life and are central to both terrestrial and prebiotic chemistry. Despite this, our understanding of how nitrogen-containing compounds like formamide and urea, along with their sulfur analogs thioformamide and thiourea, form in the cold interstellar medium (ISM) remains incomplete. The chemistry underlying their formation is largely elusive, making the elucidation of their mechanism in the ISM and EA a topic of ongoing interest. This study employs *ab initio*//density functional theory (DFT) calculations to predict the possible formation routes of amides and thioamides. The rate constants (*k*) for barrierless reactions were determined using statistical rate theory, such as microcanonical variational transition state theory (µVTST) and Rice–Ramsperger–Kassel–Marcus (RRKM)/master equation (ME) simulations, to understand their kinetic behavior. Using basic interstellar precursors—CO, CS, NH_2_, H_2_, and NH_3_—we assessed gas-phase formation routes for amides and thioamides. The data reveal that thioamides (HCSNH_2_, NH_2_CSNH_2_) may form under ISM conditions, while amides (HCONH_2_, NH_2_CONH_2_) are less likely due to their relatively high energy barriers (>5 kcal/mol). In this work, we suggest the potential detection of four new molecules in ISM environments based on enthalpy and rate constant calculations: (i) ·CSNH_2_, (ii) HCSN·H, (iii) HCSNH_2_, and (iv) NH_2_CSNH_2_. Furthermore, organosulfur-bearing molecules are identified as potential precursors to iron-sulfide grains and astrobiologically significant compounds, such as the amino acids methionine and cysteine. Understanding these mechanisms is crucial for linking the chemistries of carbon, nitrogen, oxygen, and sulfur in deep space, thereby expanding our knowledge of the sulfur cycle within the Galaxy.

## Graphical Abstract

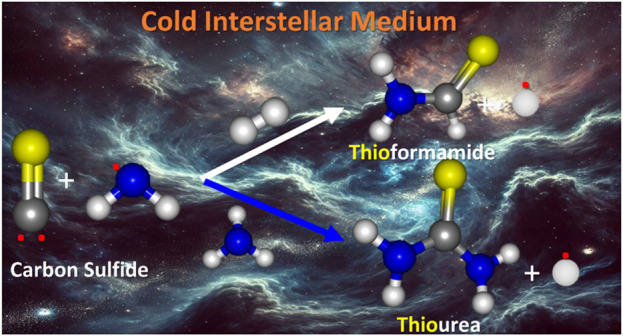

## 1 Introduction

The formation of unknown and complex chemical species in the interstellar and circumstellar envelopes (CSEs) has contributed to the limited exploration of gas-phase astrochemical compounds. More than 300 molecules have been observed in the interstellar medium (ISM), with nitrogen and sulfur-bearing species accounting for a notable portion, around 96 and 33, respectively. (Koln Database, 2024; Woon, 2004). As key components of proteins, amino acids and peptide linkages (−C(O)−NH−) are integral to terrestrial life and have become focal points in studies of prebiotic and biochemical evolution, (Damodaran and Parkin, 2017; Frenkel-Pinter et al., 2020; Awata et al., 2020; Ruiz-Mirazo et al., 2014; Wieland and Bodanszky, 2012), and have garnered significant attention in terrestrial and prebiotic chemistry. (Kubyshkin and Budisa, 2019; Ma, 2014; Das, 2022; Gomes and Rautureau, 2021; Kerkeni and Simmie, 2023; Ligterink et al., 2022). Despite the lack of confirmed amino acid detections in the ISM remains unconfirmed, several peptide-containing molecules have already been identified.

Peptide-like molecules, such as formamide (NH_2_CHO) (Rubin et al., 1971), urea [NH_2_C(O)NH_2_] (Belloche et al., 2019) acetamide (CH_3_CONH_2_) (Hollis et al., 2006), N-methylformamide (CH_3_NHCHO) (Belloche et al., 2017) and propionamide (C_2_H_5_CONH_2_) (Li et al., 2021) have been tentatively observed. Such observations indicate that peptide-containing species may be more prevalent in space than previously thought, indicating their relevance to prebiotic chemistry and life’s origins. Some of these molecules serve as precursors for the formation of adenine, guanine, cytosine, and uracil. Additionally, compounds such as urea and cyanoacetylene are promising candidates as the reactants for prebiotic cytosine synthesis.

Formamide, an important building block of life, was the first peptide-like molecule detected in space, as reported by Rubin et al. in Sagittarius B2. (Rubin et al., 1971). Despite its widespread presence in the ISM, the formation of interstellar complex organic molecules (iCOMs) like formamide remains an active area of research. Various experiments have sought to elucidate how NH_2_CHO forms, considering mechanisms occurring in the gas phase as well as on interstellar grain surfaces. (Herbst and Van Dishoeck, 2009; Charnley et al., 1992; Balucani et al., 2015; Vasyunin and Herbst, 2013; Garrod and Herbst, 2006; Öberg et al., 2009; Ruaud et al., 2015; Watanabe and Kouchi, 2002; Rimola et al., 2014). Hubbard et al. were among the first to suggest that photolysis of CO and NH_3_ under Martian atmospheric conditions could lead to NH_2_CHO formation. (Hubbard et al., 1975; Ferris et al., 1974; Kakumoto et al., 1985; Jones et al., 2011; Mason et al., 2014; Kaňuchová et al., 2016; Bredehöft et al., 2017; Dulieu et al., 2019). Kakumoto et al. analyzed the formation of NH_2_CHO in their shock tube experiments. (Kakumoto et al., 1985). Kaiser and co-workers (Jones et al., 2011) performed surface studies on formamide formation, while Mason et al. utilized electron-induced irradiation of CH_3_OH and NH_3_. (Mason et al., 2014). Strazzulla’s team carried out irradiation experiments on frozen gas mixtures (Kaňuchová et al., 2016), and Bredehöft et al. (2017) explored the formation of NH_2_CHO from mixtures carbon monoxide and ammonia. Dulieu and colleagues, on the other hand, investigated the simultaneous hydrogenation of nitric acid (HNO_3_) and formaldehyde (CH_2_O). (Dulieu et al., 2019).

The use of quantum chemical techniques has become widespread in conjunction with experimental studies, providing crucial mechanistic insights. (Oie et al., 1982; Darla and Sitha, 2019; Spezia et al., 2016; Rimola et al., 2018; Vazart et al., 2016; Barone et al., 2015). Earlier work has highlighted the formation of NH_2_CO, driven by reactions between closed-shell species, including HCOOH and NH_3._^36^ Other studies, such as those by Darla and Sitha (2019), proposed a reaction between CO and NH_3_, while Spezia et al. (Spezia et al., 2016) investigated the reaction of HCHO and ammonium hydroxide (NH_4_OH). Rimola et al. (2018) investigated various formation pathways of formamide (NH_2_CHO) on interstellar ices. They studied the radical–radical recombination reaction HCO (ice) + NH_2_ (ice) → HCONH_2_ (ice), which was found to be exothermic and barrierless, suggesting it can proceed efficiently even under the cold conditions of the interstellar medium. Their study employed a cluster model consisting of 33 H_2_O molecules to simulate water-rich amorphous ices and evaluate the atomistic mechanisms leading to formamide formation. Some theoretical works have employed a combination of radical molecule approaches. For example, Rimola et al. (Rimola et al., 2018) studied CN radical + H_2_O, while Vazart et al. (2016) and Barone et al. (2015) examined NH_2_ radicals + HCHO reaction. In another theoretical work, Enrique-Romero et al. (2019), explored radical-radical reactions, such as those between HCO and NH_2_, which suggested the formation of NH_2_CHO. Additionally, some studies investigated reactions between ionic compounds, such as NH_4_^+^ and NH_2_OH^+^ and metal ions, mediated with HCHO to form NH_2_CO. (Redondo et al., 2014; Redondo et al., 2013; Thripati et al., 2021).

Urea is another important compound with the unique characteristic of having two N–C bonds. It plays a significant role in the origin of life and serves as a precursor for the production of cytosine and uracil. (Shapiro, 1999; Menor-Salván, 2018; Saladino et al., 2004; Robertson and Miller, 1995; Wang and Bowie, 2012). Despite numerous studies on urea, many uncertainties remain regarding its formation in ISM. Previous laboratory and theoretical studies have suggested isocyanic acid (HNCO) as a possible precursor to urea. (Raunier et al., 2004). Raunier et al. (2004) were the first to propose that HNCO could serve as a building block for urea (NH_2_CONH_2_) by subjecting pure HNCO ice to vacuum ultraviolet irradiation at 10 K, which resulted in the formation of ammonium cyanate (NH_4_^+^, OCN^−^), NH_2_COH, and NH_2_CONH_2_. Another hypothesis, reported by Förstel et al. (2016), suggested that NH_2_COH could be a precursor to NH_2_CONH_2_, in which NH_3_:CO ices were initially irradiated first to produce formamide and urea. More recently, Perrero and Rimola (2024) studied the reaction between HNCO and NH_3_ on an 18 H_2_O molecule ice cluster model, which mimics interstellar ice mantles.

Despite these prior studies, the formation pathways of N-bearing molecules such as formamide and urea (amides) through chemical reactions remain largely unknown. However, the formation of S-bearing molecules, thioformamide (NH_2_CSH) and thiourea (thioamides, NH_2_CSNH_2_), is entirely unexplored and has yet to be actively investigated. Currently, no known chemical reactions have been identified that can produce thioformamide and thiourea in the ISM.

Carbon monosulfide (CS) was the first sulfur-containing molecule discovered in the ISM, identified in 1971. From a biochemical perspective, sulfur, along with H, C, O, N, and P, is considered one of the six elements for the foundation of life. (Aversa et al., 2016; Frieden, 1972; Williams, 2002; Da Silva and Williams, 2001; Sanz-Novo et al., 2024). It is found in various biomolecules, including nucleic acids, amino acids, vitamins and sugars. As suggested in the literature, two sulfur-containing amino acids -methionine (C_5_H_11_NO_2_S) and cysteine (C_3_H_7_NO_2_S)- play vital roles in protein synthesis. (Ahmad et al., 2017; Colovic et al., 2018; Stipanuk, 2020; Doddipatla et al., 2020). Additionally, organosulfur-bearing molecules are considered potential precursors to iron-sulfide grains. (Doddipatla et al., 2020).

The purpose of this study is to investigate how formamides (formamide and urea) and thioamides (thioformamide and thiourea) can form under interstellar conditions. In this context, several intriguing questions arise:

(i) How do these precursors (CO, NH_2_, NH_3_, and H_2_) undergo feasible pathways to form formamide and urea?
(ii) Can these precursors (CS, NH_2_, NH_3_, and H_2_) lead to the formation of thioformamide and thiourea ?
(iii) What are the differences in reactivity between CO and CS with the NH_2_ radical, and most importantly, how feasible are these interstellar chemical reactions?

**SCHEME 1:**
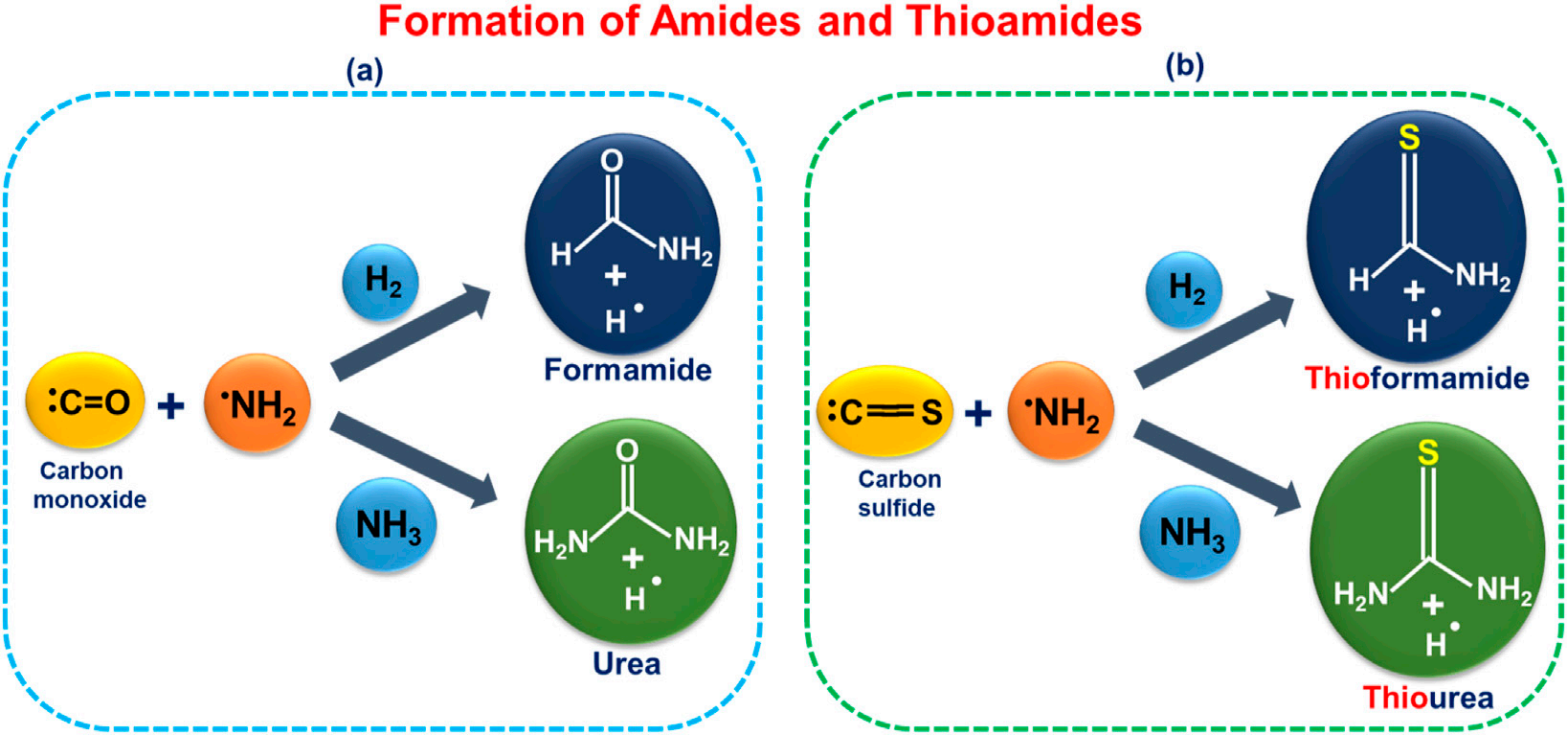
Proposed formation pathways for **(a)** amides (HCONH_2_ and NH_2_CONH_2_) (Rubin et al., 1971; Belloche et al., 2019) and **(b)** thioamides (HCSNH_2_ and NH_2_CSNH_2_) from plausible interstellar precursors—CO, CS, NH_2_, H_2_, and NH_3_. (Rickard et al., 1975; Henkel and Bally, 1985; van Dishoeck et al., 1993; Thompson et al., 1978; Cheung et al., 1968; Motiyenko et al., 2020).

In Scheme 1 below, we explore the possibility of interstellar gas-phase formation of amides (HCONH_2_, NH_2_CONH_2_) and formation of thioamides (HCSNH_2_, NH_2_CSNH_2_) through interaction between CO, CS, NH_3_, and H_2_ and the NH_2_ radical.

To investigate the formation of NH_2_CO, NH_2_CONH_2_, NH_2_CS, and NH_2_CSNH_2_, we employed *ab initio*//Density Functional Theory (DFT) calculations in combination with statistical rate theory-based predictions. This study explores an alternative stepwise mechanism involving the reaction of NH_2_ with CO, followed by H_2_ addition—a pathway that may be relevant under conditions where CO is abundant and NH_2_ is produced via ammonia photodissociation. For thioformamide and thiourea, the proposed mechanisms—based on NH_2_ + CS reactions—represent, to our knowledge, the first detailed gas-phase pathways suggested for these species under interstellar conditions. Our analysis includes structural, energetic, and kinetic details, providing key chemical insights. Finally, we discuss the astrochemical significance, of these findings and present our conclusions.

## 2 Computational details

### 2.1 *Ab initio//DFT* calculations

Gaussian 16 quantum chemical software was employed for all the *ab initio*//DFT calculations. (Gaussian 16 et al., 2016). We employed the unrestricted double-hybrid density functional method, specifically the B2PLYP functional, (Grimme, 2006), with augmented triple-zeta basis sets (Dunning, 1989) (aug-cc-pVTZ and aug-cc-pV (T+d)Z, the latter used only for the sulfur system) to optimize the structures of reactants, pre-reactive complex, intermediates, transition states, and products. The B2PLYP functional was supplemented with Grimme’s DFT-D3 zero-damping correction to address long-range London dispersion interactions. (Grimme et al., 2011). The B2PLYP-D3 double-hybrid functional is particularly well-suited for investigating noncovalent interactions in transition states, intermediates, and post-intermediates, especially in systems involving hydrogen bonding, as suggested in the earlier study. (Vazart et al., 2016). Several research groups have successfully used this method to predict the astrochemical formation of nitrogen-containing compounds such as methylamine and formyl cyanide^.^ (Tonolo et al., 2020; Puzzarini et al., 2020). To confirm the nature of the saddle point, frequency analysis was performed, revealing a single imaginary frequency for the transition states and all positive frequencies for the reactants, intermediates and products. Vibrational analysis was performed for each optimized species to include the zero-point vibrational energy (ZPE). Energy accuracy was improved by performing single-point calculations at the CCSD(T) (Raghavachari et al., 1989)/aug-cc-pVTZ basis set, using geometries optimized at the B2PLYP-D3 level. The combination of CCSD(T) and B2PLYP-D3-optimized methods has been used previously, achieving accuracy within ∼1 kcal/mol. (Ballotta et al., 2021). The zero-point energies (ZPEs) obtained using the B2PLYP-D3 functional are suitable for interstellar medium (ISM) chemistry; however, there may be an uncertainty of 1–2 kcal/mol in the computed energies, which can affect predictions for low-temperature kinetics. B2PLYP-D3 was selected over more conventional hybrid functionals because it offers a consistent and well-established balance between accuracy and computational cost, particularly for systems where both dynamical and static correlation are non-negligible. (Grimme, 2006; Dunning, 1989). The double-hybrid nature of B2PLYP, which includes a perturbative second-order correlation component, improves the description of subtle electronic effects that are critical for accurately modeling low-frequency vibrational modes and tunneling pathways. Furthermore, B2PLYP-D3 has demonstrated superior performance in reproducing vibrational frequencies and barrier heights in similar systems compared to standard hybrid functionals, especially in the presence of non-covalent interactions and anharmonic contributions. (Vazart et al., 2016; Barone et al., 2015). This aligns with the objectives of our study, which emphasize the accurate characterization of low-frequency vibrational modes associated with quantum tunneling. Earlier work by Papamokos and Demetropoulos supports the use of the hybrid functional PW91XC for amide systems and addresses low-frequency vibrational issues, as discussed in their study. (Papamokos and Demetropoulos, 2004a; Papamokos and Demetropoulos, 2004b). The increased accuracy offered by double-hybrid methods such as B2PLYP-D3 makes them particularly suitable for the current investigation, which focuses on low-temperature tunneling-relevant vibrational features and dispersion corrected zero-point energies. Our choice thus reflects a targeted effort to improve the reliability of both geometries and harmonic frequencies in a regime where conventional hybrid functionals may not be sufficiently accurate.

To investigate how the single-reference wave function qualitatively contributes, T1 diagnostic calculations were done at the CCSD(T)/aug-cc-pVTZ level. The resulting T1 diagnostic values were ≤0.03, which is within the accepted range (≤0.04) for a single-reference wave function. (Rienstra-Kiracofe et al. 2000). To evaluate spin contamination, the spin expectation value ⟨S^2^⟩ was calculated, yielding values in the range of ∼0.75–0.77, indicating that spin contamination is negligible.

### 2.2 Chemical kinetics calculations

All chemical kinetics calculations were performed using the MultiWell program suite. (Barker, 2001; Barker, 2009; Barker et al., 2016). The rate constants were calculated using RRKM/ME theory, where the energy- and angular momentum-dependent unimolecular rate coefficient, *k(E, J)* (Barker, 2001; Barker, 2009; Barker et al., 2016) is given by Equation 1:

$$k\left(E,J\right)=\frac{L^{\neq }}{h}\frac{G^{\neq }\left(E-E_{0,J},J\right)}{\rho \left(E,J\right)}$$ (1)

Here, *L* ^*+*^ is the reaction path degeneracy, *h* is Planck’s constant, *ρ(E,J)* is the rovibrational density of states of the reactant, *G* ^*+*^ *(E - E*_*0*_ *,J, J)* is the transition state sum of states, and *E*_*0*_ *,J* represents the critical energy threshold for the reaction, which is angular momentum-dependent. The reaction path degeneracy was determined from symmetry considerations and optical isomer counts. The DenSum module within MultiWell was used to compute these sums and densities of states as discussed in the MultiWell manual. (Barker, 2001; Barker, 2009; Barker et al., 2016). For efficiency, the two-dimensional terms were integrated over angular momentum to yield one-dimensional forms (Equations 2, 3):

$$G^{\neq }\left(E-E_{0,0}\right)=\sum_{J=0}^{Jmax}G^{\neq }\left(E-E_{0,J},J\right)$$ (2)

$$\rho \left(E,J\right)=\sum_{J=0}^{Jmax}\rho_{i}\left(E,J\right)$$ (3)

The 1-D rate constants *k*(*E*) were calculated as given in Equation 4:

$$k\left(E\right)=\frac{L^{\neq }}{h}\frac{G^{\neq }\left(E-E_{0,0}\right)}{\rho \left(E\right)}$$ (4)

where *E*_*0*_ *,*_*0*_ includes zero-point energy and J = 0 centrifugal corrections. Rotational motion was modeled using the symmetric top approximation (*A > B = C*), treating the *K*-rotor as active and the perpendicular 2D-rotor (*B = C*) as adiabatic. The rotational constants were derived from *B2PLYP-D3/aug-cc-pVTZ* optimized geometries and vibrational frequencies, and the Stein–Rabinovitch version of the Beyer–Swinehart algorithm was employed for statistical summations. To calculate the pressure-dependent rate constants, N_2_ bath gases were used with an approximate value of the energy transfer process $<\Delta E{>}_{down}=200\times {\left(\frac{T}{300}\right)}^{0.85}$ cm^−1^. (Ali and Balaganesh, 2023) In the MultiWell software, master equation simulations utilized double arrays containing 500 elements, each representing energy intervals of 10 cm^−1^. The quasi-continuum region was modeled up to an energy limit of 85,000 cm^-1^. Simulations at each specified temperature and pressure began with a chemical activation energy distribution, which is particularly suited for modeling recombination processes. A total of 10^6^ stochastic trials were performed, with each trial simulating a time span equivalent to the average duration of 1,000 molecular collisions. The pressure-dependent forward total rate constants, k_*f*_ for CS + NH_2_ reactions, have been computed using,

$$k_{f}=\Gamma \;K_{eq}\times k_{\infty }^{uni}\left(1-f_{\mathrm{PR}}\right)$$ (5)

The *Γ* Eckart asymmetric tunneling (EAT) correction was applied as implemented in the MultiWell master equation (ME) code, with the tight transition state explicitly calculated, *f* is the fraction of the chemical reaction going back to the respective reactive species, and $k_{\infty }^{uni}$ is the rate constant at the high-pressure limit.

The chemical kinetics of the pre-reactive complex (PRC) formation CS….NH_2_, which is assumed to play an important role in ISM condition, has not been investigated. The rate constants for CS + NH_2_ were calculated using a combination of variational transition state theory (VTST) and µVTST. (Ali and Balaganesh, 2023; Ali et al., 2016; Ali et al., 2021). The variational transition state theory (VTST) was used to calculate the rate constants as given in Equation 6 and Equation 7:

$$k^{GT}\left(T,s\right)=L^{\neq }\times \frac{k_{B}T}{h}\frac{Q_{TS}^{\neq }\left(T,s\right)}{Q_{R}\left(T\right)}\exp\left(-\frac{E_{MEP}\left(s\right)}{k_{B}T}\right)$$ (6)

$$k^{VTST}\left(T\right)=min_{s}\;k^{GT}\left(T,s\right)=k^{GT}(T,s^{VTST}\left(T\right)$$ (7)

where $k^{GT}\left(T,s\right)$ and $k^{VTST}\left(T\right)$ are the rate constants of generalized and variational transition state theory, respectively, $E_{MEP}$ is the zero-point corrected barrier height. Based on VTST calculations, µVTST calculations were carried out as discussed in our previous work. (Ali and Balaganesh, 2023; Ali et al., 2016; Ali et al., 2021) The *ktools* module within the MultiWell program was used to perform the VTST and µVTST calculations, as described in the MultiWell manual. (Barker, 2001; Barker, 2009; Barker et al., 2016) The equilibrium constant (*K*_*eq*_) for the formation of bimolecular complexes were calculated using THERMO code as given in Equation 8:

$$K_{eq}=\frac{Q_{\mathrm{Int}}}{Q_{R}}\exp\left(-\frac{E_{\mathrm{Int}}-E_{R}}{k_{B}T}\right).$$ (8)

## 3 Results and discussion

Here, we initially highlight our computational findings on the formation of formamide, urea, thioformamide, and thiourea under interstellar conditions. Consistent with the previous work research, (Thripati et al., 2021; Thripati and Ramabhadran, 2017; Gopalsamy et al., 2019; Thripati, 2022; Thripati et al., 2023)^,^ we used zero-point corrected energies rather than free energies. This approach prevents any inferences based on the predominance of Boltzmann distributions, which may not consistently hold true in all regions of the ISM. Additionally, this study references all energies, including those of minimized energy structures and transition states, relative to the energy of individual monomers when they are infinitely separated, which is considered the baseline energy of zero. Our previous investigation employed a similar paradigm, which remains valid for gas-phase processes occurring in the ISM under low-density conditions. Therefore, the negligible significance of collisional deactivation—more relevant to solution-phase processes on Earth—supports our selection of the zero-reference state.

### 3.1 Methods validation

Barrier heights are important parameters for determining astrochemical reactions in the ISM. To ensure consistency with other theoretical methods, several *ab initio* and DFT calculations (B2PLYP-D3 (Grimme, 2006; Grimme et al., 2011), B3LYP (Becke, 1993; Lee et al., 1988), M06-2X (Zhao and Truhlar, 2008), and ωB97XD (Chai and Head-Gordon, 2008)) were performed using the CCSD(T)/aug-cc-pVTZ//B2PLYP-D3/aug-cc-pVTZ, CCSD(T)/aug-cc-pVTZ//B3LYP/aug-cc-pVTZ, CCSD(T)/aug-cc-pVTZ//M06-2X/aug-cc-pVTZ, and CCSD(T)/aug-cc-pVTZ//ωB97XD/aug-cc-pVTZ methods for both amide and thioamide schemes. A combination of CCSD(T) and several DFT-based optimization methods has been successfully used in our past work, showing good consistency with experimental findings for similar systems. (Ali and Balaganesh, 2022; Ali et al., 2019; Ali et al., 2018; Ali, 2020; Ali et al., 2022; Ali, 2019; Ali and Saswathy, 2024; Ali and Barker, 2015). The results in Table 1 show that our calculations align with different levels of theory, with a deviation of less than 0.5 kcal/mol for the pre-reactive complex and transition state. The energy analysis in Table 1 clearly indicates that CCSD(T)//B2PLYP-D3 values are accurate compared to the other three DFT methods. As suggested in previous work, (Puzzarini et al., 2020), CCSD(T)//B2PLYP-D3 provides accurate energies compared to the “Cheap” composite method. Therefore, all minimum energy pathway (MEP) and kinetics calculations were performed at the CCSD(T)//B2PLYP-D3 level.

**TABLE 1:** Comparison of the energy barriers ΔE^≠^ (kcal/mol) with different level of theories on representative TSs with respect to Reactants.

Formamide and urea	^a^CCSD(T)//B2PLYP-D3	^a^CCSD(T)//ωB97XD	^a^CCSD(T)//B3LYP	^a^CCSD(T)//M06-2X
TS-1	5.5	5.4	5.2	5.5
TS-2	−0.4	−0.5	−0.6	−0.8
TS-3	34.2	34.4	34.1	34.3
TS-4	12.1	12.3	12.2	12.1
TS-5	13.4	13.8	13.4	13.5
TS-6	22.1	22.3	21.9	22.2

Thioforamamide and Thiourea	^b^CCSD(T)//B2PLYP-D3	^b^CCSD(T)//ωB97XD	^b^CCSD(T)//B3LYP	^b^CCSD(T)//M06-2X
TS-1_s_	−1.4	−1.49	−1.4	−1.7
TS-2_s_	−35.9	−36.2	−36.1	−36.2
TS-3_s_	−3.3	−3.21	−3.5	−3.4
TS-4_s_	−27.1	−27.4	−27.0	−27.7
TS-5_s_	−25.7	−25.8	−25.8	−26.1
TS-6_s_	−26.3	−26.2	−26.4	−26.6

Where ΔE^≠^ = E_TS_– $E_{\infty }$ separated reactants. The energy values (including zero-point corrections) are provided in kcal/mol. basis set a:aug-cc-pVTZ; b: aug-cc-pV (T+d)Z.

To further validate our results on the formation of PRC in the sulfur system, we employed several quantum composite methods, including CBS-QB3 (Montgomery et al., 2000), G4 (Curtiss et al., 2007), and W1BD (Barnes et al., 2009) methods were employed (see ESI Supplementary Figure SI,13). These calculations indicate that our results are consistent across different levels of theory, with a difference of less than 0.5 kcal/mol for the pre-reactive complex and transition state. Therefore, we believe the results presented in this paper are reasonably accurate for astrochemical implications.

### 3.2 Formation of amides [–(C=O)–NH–]

**SCHEME 2:**
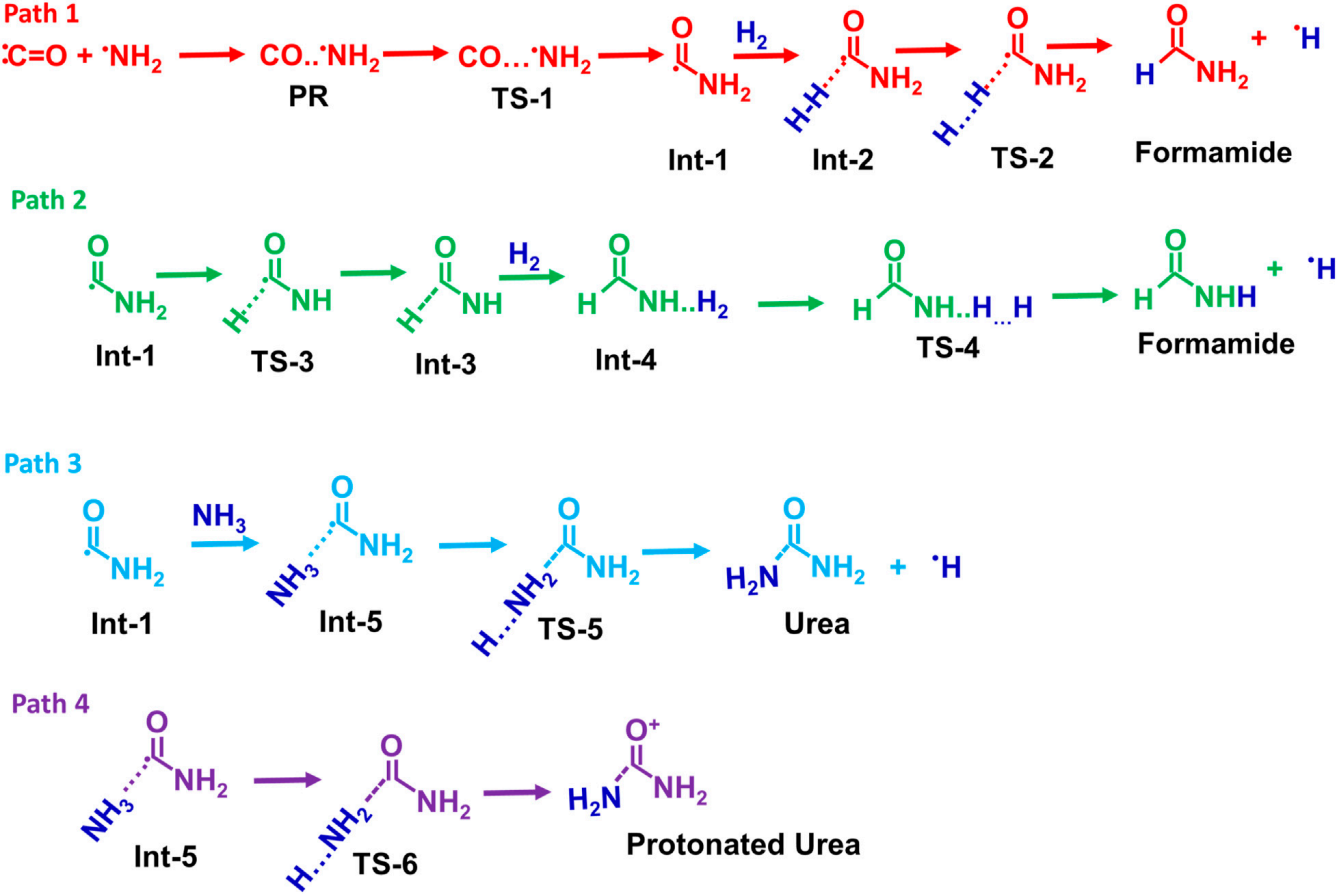
Possible reaction pathways for the formamide (Path 1 and Path 2), urea (Path 3), and protonated urea (Path 4).

Possible reaction pathways for the formamide (Path 1 and Path 2), urea (Path 3), and protonated urea (Path 4) are shown in Scheme 2.

**FIGURE 1:**
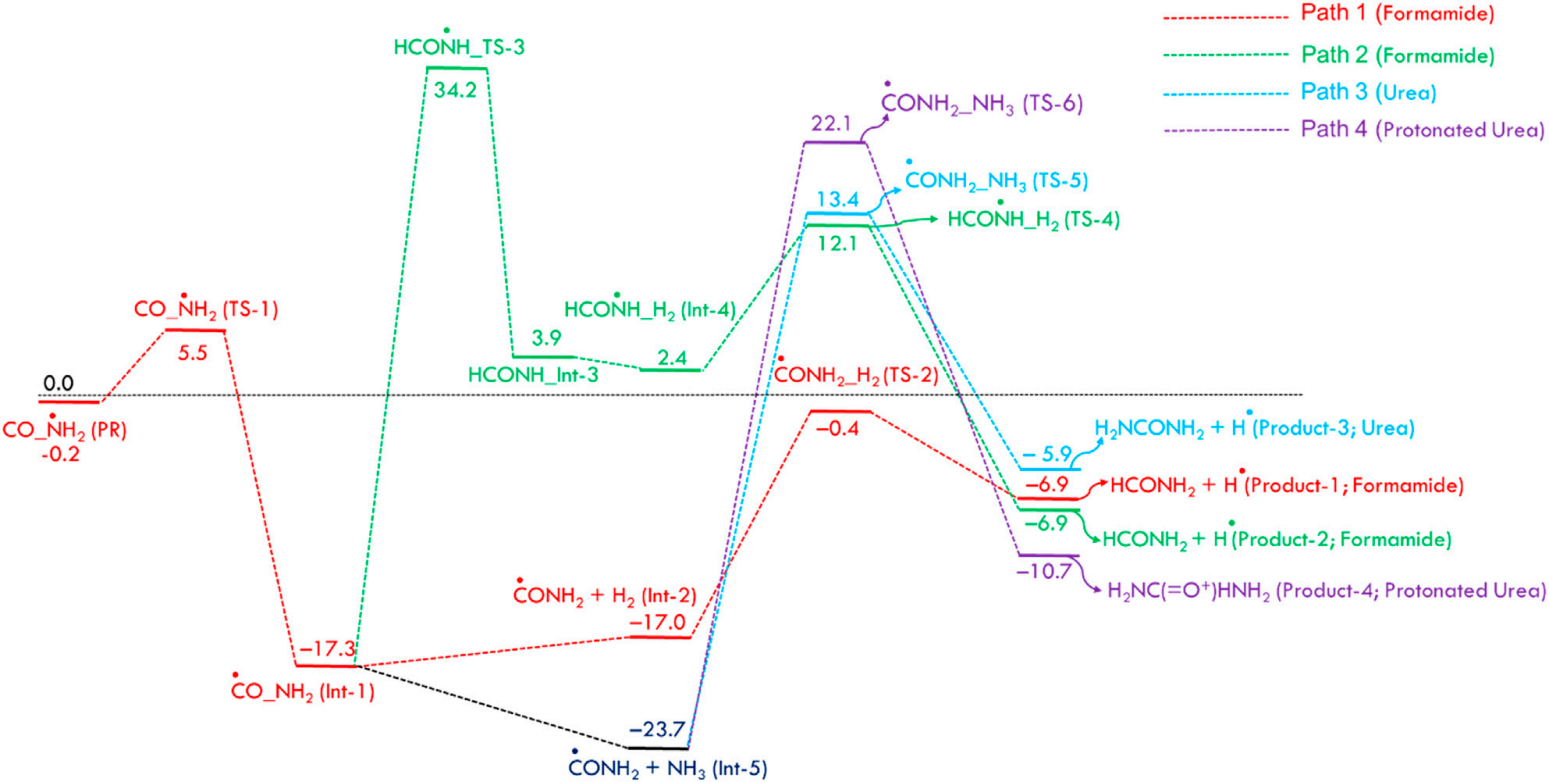
Zero-point corrected potential energy surface (in kcal/mol) for the formation of formamide and urea from CO, NH_2_, H_2_, and NH_3_, computed at the CCSD(T)/aug-cc-pVTZ//B2PLYP-D3/aug-cc-pVTZ level of theory.

Figure 1 displays the PES for formamide and urea formation. The optimized structures of reactants, pre-reactive complex, intermediates, transition states and products, along with energy values, are shown in ESI Supplementary Figure S1. The Cartesian coordinates for all these species are tabulated in ESI Supplementary Table S1. When the ^·^NH_2_ radical attacks the carbon atom of CO, a pre-reactant complex (OC· ··NH_2_) is formed (see ESI Supplementary Figure S1). The computed energy for the formation of PRC is nearly −0.2 kcal/mol (at CCSD(T)/aug-cc-pVTZ//B2PLYP-D/aug-cc-pVTZ), which is in good agreement with the energy computed energy at CCSD(T)/aug-cc-pVTZ//ωB97X-D/aug-cc-pVTZ level. This result is attributed to a weak interaction between C and N atoms. To form a C–N bond between N of ^·^NH_2_ and C of CO, both molecules come closer to each other via a transition state (TS-1), incurring a significant energy penalty. The barrier height for C-N bond formation is approximately ∼6 kcal/mol, leading to the formation of the intermediate Int-1 (^·^CONH_2_). The energy value is well-supported by calculations performed at the CCSD(T)/aug-cc-pVTZ level using ωB97X-D optimized geometries. The Int-1 (^·^CONH_2_) can react with hydrogen molecules to form Int-2 (H-H … CONH_2_), with an energy barrier of 16.6 kcal/mol, ultimately dissociates via transition state TS-2 to produce formamide (HCONH_2_) + H. As shown in path-2, Int-1 can undergo isomerization via H migration, forming Int-3 (HCONH) through a transition state (TS-3), which has a high barrier of 51.5 kcal/mol relative to Int-1 (^⋅^CONH_2_). Additionally, the transition state (TS-4) involves the addition of hydrogen molecules to form Int-4 (H-H … CONH_2_). Due to the high energy barrier and the formation of endothermic intermediates, Path-2 is less feasible compared to Path-1, making such a reaction more relevant under combustion conditions rather than ISM environments.

As shown in Figure 1, the ^·^NH_2_ radical reacts with CO to form Int-1 (^·^CONH_2_), which then reacts with H_2_ to produce formamide (HCONH_2_), following a sequential two-body process. In this study, we consider that in the presence of H_2_, CO, and ^·^NH_2_ molecules, simultaneous three-body collisions are highly unlikely. Instead, the reaction is expected to proceed through the formation of two-body complexes, which subsequently collide with a third species to form three-body complexes and products. A sequential two-body collision mechanism is therefore proposed, as illustrated in *ESI Page S18*. Such reaction hypotheses for the ISM and EA will be validated through kinetic calculations and *vice versa.*

To understand the formation of urea (NH_2_CONH_2_), Int-1 can also react with NH_3_ molecules to form CONH_2_· ·NH_3_ (Int-5), which is more stable than Int-1. This increased stability is due to the formation of two hydrogen bonds from ammonia molecules (see ESI Supplementary Figure SI,1). As shown in Figure 1, the Int-5 involves two pathways, Path 3 and Path 4, to generate NH_2_CONH_2_ molecules. Path 3 involves the formation of a second C-N bond of Int-5, which then dissociates via transition state TS-5, leading to the production of NH_2_CONH_2_ and H radical with a barrier of 37.1 kcal/mol. Another possibility involves the simultaneous formation of a C – N bond and protonation of CONH_2_, resulting in the formation of the protonated urea (Product-4). The increased stability of this product is due to the formation of three hydrogen bonds. Based on the barrier height calculations, which are relatively high in all four proposed pathways, we believe the formation of NH_2_COH and NH_2_COH_2_ is unlikely to occur in the gas-phase ISM.

### 3.3 Formation of thioamides [–(C=S)–NH–]

**SCHEME 3:**
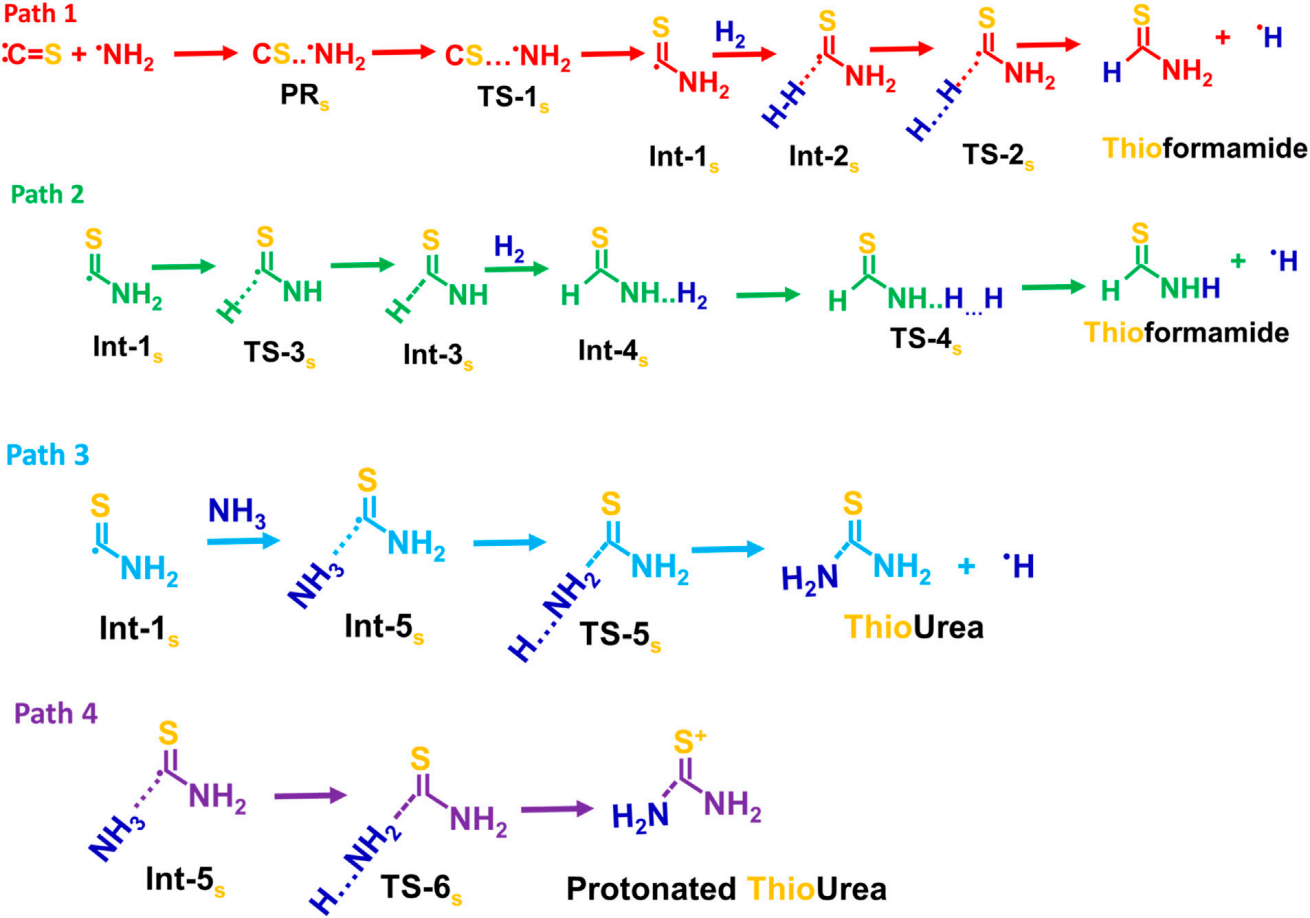
Possible reaction pathways for the thioformamide (Path 1 and 2), thiourea (Path 3), and protonated thiourea (Path 4).

Possible reaction pathways for the thioformamide (Path 1 and 2), thiourea (Path 3), and protonated thiourea (Path 4) are shown in Scheme 3.

**FIGURE 2:**
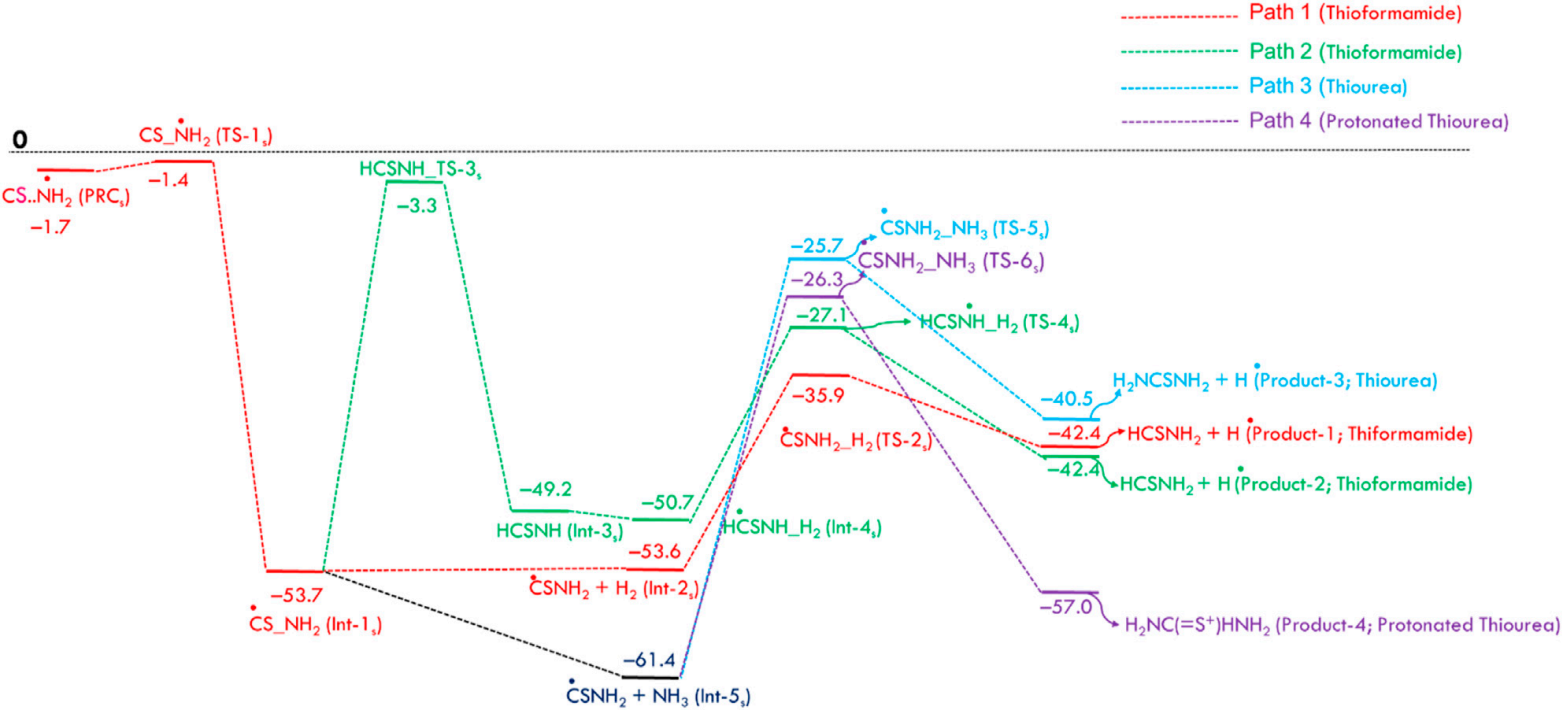
Zero-point corrected potential energy surface (in kcal/mol) for the formation of thioformamide and thiourea from CS, NH_2_, H_2_, and NH_3_, computed at the CCSD(T)/aug-cc-pV (T+d)Z//B2PLYP-D3/aug-cc-pV (T+d)Z level of theory.

Figure 2 displays the PES for the formation of thioformamide (NH_2_CSH) and thiourea (NH_2_CSNH_2_), including zero-point energy corrections. Carbon sulfide (CS) is the S-analog of carbon monoxide (CO), with the primary difference between them arising from their dipole moments. The C=S (μ = 1.912 D) has a significantly higher dipole moment than the C=O (μ = 0.113 D), which is reflected in the longer bond length of CS (1.52 Å) than CO 1.12 Å. The formation of a pre-reactant complex (CS····NH_2_, PRC_s_) is a barrierless process with an energy of −1.7 kcal/mol (w.r.t. Reactants). This stationary point is 0.3 kcal/mol lower than that of the transition state TS-1_s_ to form a submerged barrier relative to the reactants at infinite separation, facilitating N-S bond formation. This is because carbon sulfide has a higher dipole moment, bond length, and polarizability, leading to more reactivity with NH_2_ radicals.

The Int-1_s_ (^·^CSNH_2_) can react with hydrogen molecules to form Int-2_s_ (H_2_ … CSNH_2_) which then dissociates via transition state TS-2_s_ to produce thioformamide (HCSNH_2_) + H radical, with an energy barrier of 17.7 kcal mol^-1^, as seen in Path 1. In Path 2, Int-1 (see ESI Supplementary Figure S2) undergoes isomerization to form Int-3_s_ (HCSN·H), which then undergoes hydrogenation, followed by breaking the H-H bond, resulting in the formation of thioformamide and a H radical. As shown in ESI Supplementary Figure SI,2, Path 3 and Path-4 lead to two different transition states (TSs) for thiourea (TS-5_s_) and protonated thiourea (TS-6_s_). In this process, ammonia reacts with C of Int-1_s_, forming thiourea and a H radical. Another possible reaction pathway (Path 4) involves Int-5_s_ reacting with an ammonia molecule, simultaneously leading to C–N bond formation and protonation (H^+^) of Int-1_s_. This reaction results in the formation of protonated thiourea, which is more stable than thiourea. As shown in Figure 2, since the first barrier is low, the other barriers are relatively lower, and the exothermic nature of the products, Path 1, Path 3, and Path 4, is likely to be feasible under ISM conditions. On the other hand, Path 2 has a barrier height of ∼50 kcal/mol, making this pathway infeasible under ISM conditions.

The dipole moment is a crucial parameter for detecting molecules in the ISM, as it can be analyzed using microwave spectroscopy (Kroto, 1981) and quantum chemical calculations. Molecules with smaller dipole moments exhibit weak emissions and are difficult to detect, even if they are abundant in the ISM. The dipole moments (in debye) for stable amide species are as follows: CO (0.113), CONH_2_ (3.693), HCONH (3.388), HCOONH_2_ (3.975), and NH_2_CONH_2_ (4.384). For stable thioamide species, the values are CS (1.912), Int-1_s_ (^·^CSNH_2_) (3.786), Int-3_s_ (^·^HCSNH) (2.209), thioformamide (HCSNH_2_) (4.528), and thiourea (NH_2_CSNH_2_) (5.586). Molecules with higher dipole moments are more likely to be detected in the ISM. Therefore, sulfur-containing compounds such as Int-1_s_ (^·^CSNH_2_), thioformamide (HCSNH_2_), and thiourea (NH_2_CSNH_2_) may also be present. However, experimental evidence is needed to confirm this prediction.

### 3.4 Rate constants for CS + NH_2_ reaction

**FIGURE 3:**
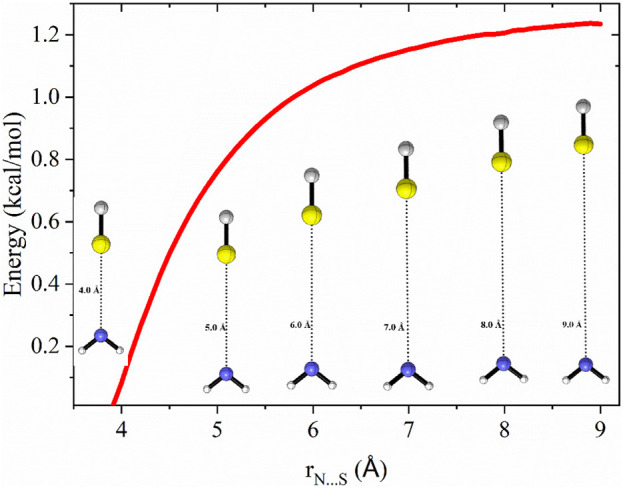
Zero-point corrected potential energy surface (in kcal/mol) as a function of bond distance for the dissociation of CS⋯NH_2_ → CS + NH_2_, computed at the CCSD(T)/aug-cc-pVTZ//B2PLYP-D3/aug-cc-pVTZ level of theory.

To identify the transition state for the CS⋯NH_2_ → CS + NH_2_ dissociation, the minimum energy path (MEP) was computed by performing constrained optimizations along the bond distance, incorporating zero-point energy corrections for the vibrational modes orthogonal to the forming bond, as illustrated in Figure 3. The MEP was determined using the B2PLYP-D3/aug-cc-pVTZ level of theory, performing constrained optimization at S-N distances ranging from 3.8 Å to 9.0 Å in increments of 0.1 Å.

**FIGURE 4:**
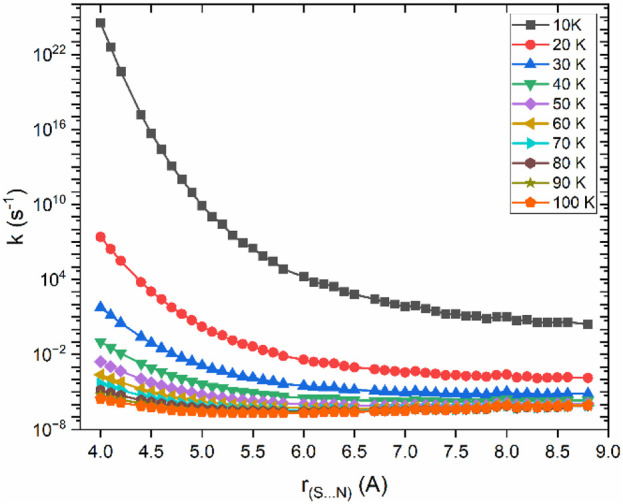
Rate constants as a function of bond distance for the dissociation of CS⋯NH_2_ → CS + NH_2_, calculated at the CCSD(T)/aug-cc-pVTZ//B2PLYP-D3/aug-cc-pVTZ level of theory.

The reaction path illustrated in Figure 3 was used to calculate VTST rate constants across a temperature range of 10 K–100 K are shown in Figure 4.

**FIGURE 5:**
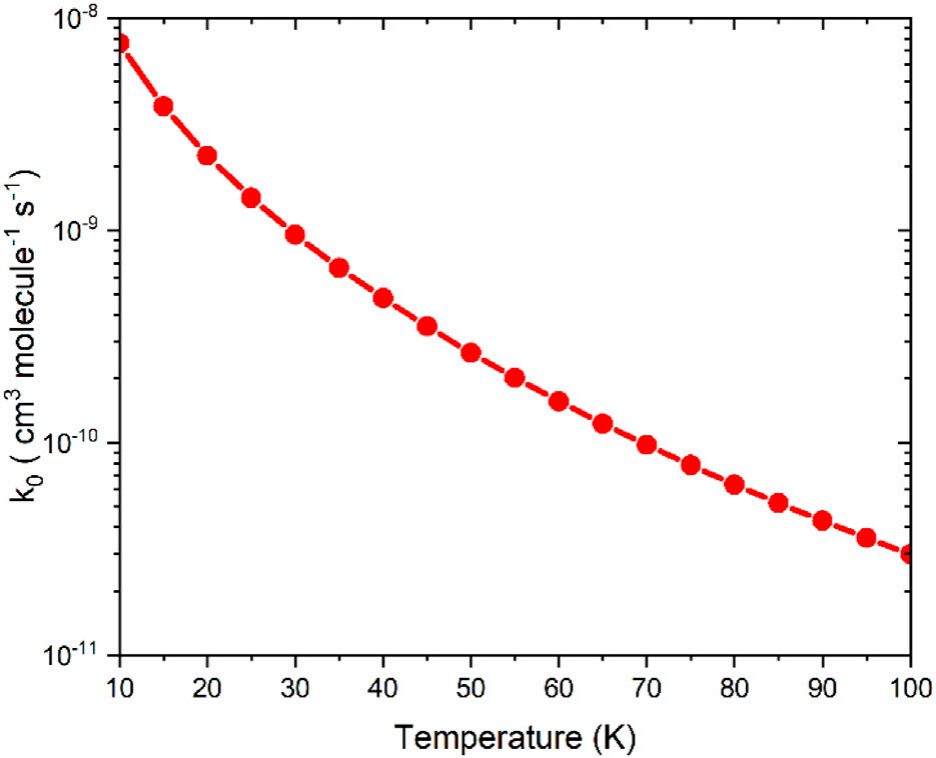
The capture rate constants associated with the entrance channels were computed using the microcanonical variational transition state theory method.

Based on the VTST calculation, the µVTST rate constants range from approximately 10^–8^ to 10^–11^ cm^3^ molecule^−1^ s^-1^ between 10K and 100K, as shown in Figure 5. The tabulated rate constant values are provided in ESI Supplementary Table SI,7. As highlighted in earlier research, barrierless reactions can exhibit a negative temperature dependence, which supports a capture-type mechanism facilitated by long-range intermolecular forces. These forces grow more influential at lower temperatures as thermal energy diminishes. Our results reflect a similar trend, comparable to the NH_2_ + NO reaction. (Klemperer, 2006).

**FIGURE 6:**
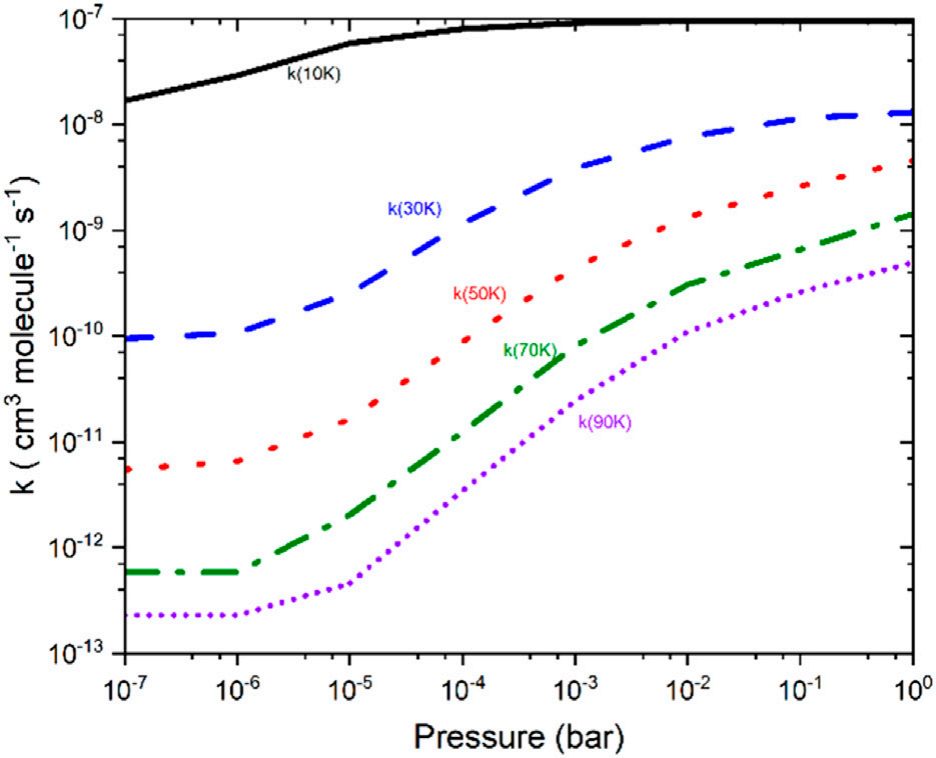
Temperature- and pressure-dependent total rate constants for the CS + NH_2_ → Int-1_s_ reaction were calculated using RRKM/master equation (RRKM/ME) simulations.

Figure 6 presents the rate constants for the CS + NH_2_ reaction over a pressure range from 10^–7^ bar–1 bar at various temperatures. The figure reveals that the greatest disparity between the two limits occurs around 90 K, with a difference of nearly three orders of magnitude. In contrast, at 10 K, the difference between the two regimes is approximately a factor of 5. To gain a deeper understanding of the formation of thioformamide and thiourea, the rate constants for these channels were also calculated for temperatures ranging from 10 K to 30 K at a very low pressure of approximately 10^–10^ bar. Our calculations show that at low temperatures (<30 K) and very low pressures, Int-1_s_ forms efficiently. However, at high pressures (>10^–7^ bar) and temperatures (>30 K), the reaction tends to revert to the reactants, making the formation of thioformamide and thiourea nearly negligible. Our analysis suggests that thioformamide (NH_2_CS) and thiourea (NH_2_CSNH_2_) can be formed from Int-1_s_ + H_2_ and Int-1_s_ + NH_3_, respectively, under conditions of very low temperature and pressure. We also considered the role of competitive reactions, such as Radiative Association (RA), which are significant in interstellar medium (ISM) conditions. The forward reaction rates at low temperatures and very low pressures are faster than typical RA reactions, which generally exhibit rate constants well below ∼10^–11^ cm^3^ molecules^-1^ s^-1^. In other words, the reactive intermediate (Int-1_s_) will interact with “third body” species such as H_2_ or NH_3_, leading to the formation of NH_2_CSH and NH_2_CSNH_2_. From the data in Figure 6, the low-pressure analysis (10^–7^ bar, typical of experimental conditions) shows that under ISM-like pressures (P < 10^–14^ bar, corresponding to a molecular density of approximately 10^6^ molecules/cm^3^), the reactive intermediate (Int-1_s_) can still form effectively. This is because the pressure effect is almost negligible.

### 3.5 Chemistry of ISM

In the case of formamide (HCONH_2_) and urea (NH_2_CONH_2_) formation, all the precursor molecules, such as CO, ^·^NH_2_, H_2_, and NH_3_, have been detected in ISM. However, all transition states (TS-1 to TS-6) involved in Path 1 and Path 2 (formamide formation), Path 3 (urea formation), and Path 4 (protonated urea formation) are infeasible due to the energy barriers. For thioformamide and thiourea formation, it is important to note that the precursors used in this study, CS, ^·^NH_2_, H_2_, and NH_3,_ have been observed in the ISM. However, intermediates such as Int-1_s_ (^·^CSNH_2_) and Int-3_s_ (HCSN·H), as well as products like thioformamide (HCSNH_2)_ and urea (NH_2_CSNH_2_), have not yet been detected. The transition states (TS-1_s_ to TS-6_s_) involved in Path 1 (thioformamide formation), Path-3 (thiourea formation), and Path 4 (Protonated thiourea formation) are feasible under interstellar conditions due to their lower barrier heights and kinetic feasibilities (see Figures 2, 6).

Following the approach suggested in previous studies (Ballotta et al., 2021; Klippenstein et al., 1996), the effective association reaction in the low-pressure limit was calculated as $k_{eff}=\frac{k_{f}\;k_{r}}{k_{b}+k_{r}}$ , where k_*f*_ is the forward association rate constant (cm^3^ molecule^−1^ s^-1^), k_r_ (s^-1^) is the radiative stabilization rate constant and k_b_ (s^-1^) is the back-dissociation rate constant. To assess the accuracy of radiative stabilization, the forward rate constant was calculated using the µVTST_RRKM/ME method, as discussed in section 3.4. k_f_ varies from 10^–8^ cm^3^ molecule^−1^ s^-1^ to 10^–11^ cm^3^ molecule^−1^ s^-1^ from the temperature range of 10–100K. As suggested in previous studies (Dalgarno, 1987; Van Dishoeck, 2014; Douglas et al., 2024), and anticipated in our calculation, the k_r_ is expected to be higher than the backward reaction (k_b_). In that case, the effective association rate for this process is almost expected to be the same as the forward reaction (k_r_ >> k_b_, the $k_{eff}=kf).$ This analysis also agrees with the literature value of similar ISM product formation under low temperature and low-pressure conditions, which is in the range of 10^–9^ to 10^–11^ cm^3^ molecule^−1^ s^-1^. (Raghavachari et al., 1989; Klippenstein et al., 1996; Dalgarno, 1987).

The above analysis is further supported by the high dipole moments of each species in the sulfur system. The ISM is characterized by extremely low density (10^2^–10^6^ atoms, molecules, and ions per cm^3^) and temperatures ranging from 10K to 100 K). (Klemperer, 2006). Due to these conditions, chemical reactions in the ISM are typically barrierless (or involve submerged TSs) and lead to exothermic products. (Van Dishoeck, 2014; Douglas et al., 2024; Jensen, 2007). On Earth, gas phase reactions typically involve the formation of pre-reactive complexes through weak interactions between reactants, often via two-body collisions, with occasional involvement of a third body. However, in interstellar chemistry, three-body collisions are highly improbable due to the extremely low number density, cold temperatures, and long reaction time scales. (Herbst, 1985). For a dense interstellar cloud with a number density of 10^5^ cm^−3^, the typical chemical reaction timescales are: reactions involving “inert” neutral species ∼10^5^ years (or even longer in some cases), reactions of reactive neutral species ∼100 years, reactions of molecular ions, 1 hour–100 years. This aspect has been well-documented in the literature for several years. (Herbst and Klemperer, 1976).

We estimated the timescale for the formation of Int-1s using an approximate association rate of k∼10^–8^ to 10^–13^ cm^3^ s^-1^ over a temperature range of 10–100 K, under extremely low-pressure conditions. from 10 K to 100K at extremely low pressure for the formation of Int-1s. These calculations assume particle densities ranging from 10^2^ to 10^6^ cm^−3^, representative of diffuse and molecular clouds.”.

At 10 K, τ∼1/(k⋅n_reactant)_ = (10^–8^ cm^3^ s^-1^*10^2^ cm^-3^) = 10^6^ sec = ∼ 12 days.

At 100K τ∼1/(k⋅n_reactant)_ = (10^–13^ cm^3^ s^-1^*10^6^ cm^-3^) = 10^7^ sec = 115 days.

Based on the above facts, we believe the reaction mechanism proposed in this work aligns with the current understanding of interstellar gas-phase chemistry.

### 3.6 Astrochemical implications and limitations

We explicitly acknowledge that, to date, neither thioformamide nor thiourea has been detected in the interstellar medium (ISM). Although our quantum chemical and kinetic calculations indicate that their formation is feasible under cold gas-phase conditions, there is currently no observational spectroscopic confirmation. This limitation is now clearly emphasized, and our study is framed as a theoretical foundation to motivate targeted astronomical searches—particularly in sulfur-rich interstellar environments. Our proposed reaction mechanisms assume either radiative stabilization or sufficiently long-lived intermediates to permit subsequent reactions under the ultra-low-pressure conditions of the ISM. While we apply RRKM/master equation (RRKM/ME) simulations where applicable, significant uncertainties remain, especially in estimating radiative association rates and the stability of reactive complexes. These assumptions are now explicitly discussed, and we underscore the need for both experimental and theoretical investigations into the dynamics and energetics of such stabilization pathways. We note that ion–molecule and grain-surface reactions are known to play crucial roles in the formation of prebiotic species such as formamide. While our current work focuses exclusively on neutral–neutral gas-phase pathways, we now recognize the potential importance of alternative routes, including ion-mediated reactions and grain-surface processes such as HCO + NH_2_ coupling on dust grains. Given the large permanent dipole moments predicted for both thioformamide and thiourea, we strongly recommend laboratory rotational spectroscopy studies to provide reference spectra for radioastronomical detection. Finally, we highlight the need for astrochemical modeling using our computed rate constants to evaluate the viability and relative importance of these proposed formation pathways across a range of ISM environments.

## 4 Conclusion

This study provides a comprehensive theoretical investigation into the gas-phase formation pathways of amides and thioamides in the interstellar medium (ISM), employing high-level quantum chemical methods [CCSD(T)//B2PLYP] along with µVTST and RRKM/ME simulations. While reaction pathways for formamide and urea formation via NH_2_ + CO and subsequent hydrogenation and ammoniation appear unfavorable in the gas phase due to significant energy barriers, alternative routes on interstellar ices remain promising and warrant further exploration. In contrast, the formation of thioamides such as thioformamide (HCSNH_2_) and thiourea (NH_2_CSNH_2_) emerges as kinetically and thermodynamically viable under ISM conditions, with exothermic and largely barrierless reaction profiles. Importantly, we propose the existence and potential detectability of four novel sulfur-containing species—·CSNH_2_, HCSN·H, HCSNH_2_, and NH_2_CSNH_2_—which have not yet been conclusively observed in the ISM. Among these, thioformamide and thiourea are highlighted as particularly promising due to their feasible gas-phase formation pathways and strong binding energies. This study presents, for the first time, detailed gas-phase mechanisms for these molecules, especially involving NH_2_ + CS and urea-thione analogues, emphasizing their astrochemical relevance. Our findings serve as a foundation for future astronomical searches, laboratory spectroscopic studies, and astrochemical modeling efforts focused on sulfur-bearing prebiotic molecules. By improving our understanding of sulfur chemistry in the ISM, this work contributes to the broader quest to unravel the molecular origins of life and the chemical complexity of the universe.

## 5 Supporting Information

The Cartesian coordinates of formamide, urea, thioformamide, and thiourea are provided for the pre-reactant, transition state (TS), and products. The optimized geometries and their energy barriers are compared using different DFT methods on representative TSs. The study explains how different sequences of two-body reactions lead to the same highly stabilized pre-reaction complex. The energy differences between the products and reactants of formamide, urea, thioformamide, and thiourea are analyzed. Additionally, the TS energy barriers of formamide, urea, thioformamide, and thiourea are compared using different methods for representative molecules. The electronic energies for all investigated compounds (in Hartrees) are reported. The Cartesian coordinates of formamide, urea, thioformamide, and thiourea are provided for the pre-reactant, transition state (TS), and product complex. ZPE corrected energies of PRC and TS-1 for CS + NH2 at different levels of theory. The equilibrium constant and rate constants (in cm^3^ molecule^−1^ s^-1^) for the reaction CS + NH_2_ → CS … NH_2_ is also presented.

## Data Availability

The original contributions presented in the study are included in the article/Supplementary Material, further inquiries can be directed to the corresponding author.
