# Supplementary material for: Computational prediction for the formation of amides and thioamides in the gas phase interstellar medium
Source: Front Chem. 2025 Jun 30;13:1615586. doi: 10.3389/fchem.2025.1615586 (PMC12257780; doi:10.3389/fchem.2025.1615586)
Supplement: Supplementary file 1 [file DataSheet1.docx]

**Electronic Supplementary Information**

**Computational Prediction for the Formation of Amides and Thioamides in the Gas Phase Interstellar Medium (ISM)**

**^a,b^Mohamad Akbar Ali* and ^a,b^Sorakayala Thripati,**

^a,b^ Department of Chemistry, Khalifa University of Science and Technology, P.O. Box 127788, Abu Dhabi, UAE

^a,b^Center for Catalysis and Separations, Khalifa University of Science and Technology, P.O. Box 127788, Abu Dhabi, UAE

**Emails**: [akbar.mohamad@ku.ac.ae](mailto:akbar.mohamad@ku.ac.ae)

**List of Contents**

1. The Cartesian coordinates of formamide, urea, thioformamide, and thiourea are provided for the pre-reactant, transition state (TS), and product complex. Additionally, the magnitude of the imaginary frequencies (only for transition states), charge, and multiplicity are included. *Page S2- S28*
2. Comparing the optimized geometries with their energy barriers with different DFT methods on representative TSs. *Page S29- S35.*
3. Explaining how different sequences of two body reactions lead to the same highly stabilized pre-reaction complex. *Page S36- S38.*
4. The energy difference between products and reactants of Formamide, Urea, Thioformamide, and Thiourea. *Page S39.* Table SI_1-SI_4.
5. Comparing the Formamide, Urea, Thioformamide, and Thiourea TSs imaginary frequency with different methods for representative molecules. *Page 40.* Table_SI_5
6. The electronic energies for all investigated compounds (in Hartrees) *Page S41*Table_SI_6.
7. The Equilibrium constant and rate constants (in cm^3^ molecule^-1^ s^-1^) for CS+NH_2_🡺 CS…NH_2_*. Page S42* Table_SI_7.
8. Zero-point corrected energies of pre-reactive complex and TS-1 in the case of CS+NH_2_ reaction at different levels of theories *Page S43*

The Cartesian coordinates of formamide, urea, thioformamide, and thiourea are provided for the pre-reactant, transition state (TS), and product complex. Additionally, the magnitude of the imaginary frequencies (only for transition states), charge, and multiplicity are included.

The sequence of the coordinates presented here corresponds to the order provided in the main text.

The same level of theory as given in the main text is used. If an imaginary frequency is not specified, then the structure corresponds to an optimized minimum energy structure.

**Table S1**. The XYZ coordinates and Normal modes of frequencies of formamide, urea, thioformamide, and thiourea are provided for the pre-reactant, transition state (TS), and product complex.

**CO**

XYZ coordinates

C 0.00000000 0.00000000 -0.64611154

O 0.00000000 0.00000000 0.48527854

Vibrational frequency (cm-1)

*2153.72 cm^-1^*

**NH_2_**

XYZ coordinates

N 0.000000 0.141135 0.000000

H 0.804043 -0.493971 0.000000

H -0.804043 -0.493971 0.000000

Vibrational frequency (cm^-1^)

*1540.87*

*3388.48*

*3483.85*

**H_2_**

XYZ coordinates

H 0.000000 0.000000 0.370069

H 0.000000 0.000000 -0.370069

Vibrational frequency (cm^-1^)

*4461.05*

**NH_3_**

XYZ coordinates

N 0.000000 0.000000 0.114051

H -0.000000 0.939797 -0.261140

H 0.813888 -0.469898 -0.261140

H -0.813888 -0.469899 -0.261140

Vibrational frequency (cm^-1^)

*1037.05*

*1673.38*

*1673.38*

*3488.66*

*3616.94*

*3616.95*

**CO…NH_2__Pre-Reactant**

XYZ coordinates

C 0.902766 0.530918 -0.000905

N -1.983228 -0.213563 0.000534

H -2.164963 0.398841 0.801145

H -2.165928 0.393791 -0.803698

O 1.656727 -0.312287 0.000512

Vibrational frequency (cm^-1^)

*36.59*

*55.43*

*94.70*

*121.12*

*219.13*

*1543.28*

*2155.39*

*3394.38*

*3488.04*

**CO…NH_2__TS-1**

XYZ coordinates

C -0.480022 0.478956 0.000506

N 1.463911 -0.228645 -0.000085

H 1.786136 0.320978 -0.801385

H 1.786110 0.319589 0.802175

O -1.361898 -0.240574 -0.000413

Vibrational frequency (cm^-1^)

***301.63i***

*48.29*

*301.68*

*652.15*

*747.29*

*1544.37*

*2080.38*

*3399.04*

*3492.68*

**CO…NH_2__Int-1**

**XYZ coordinates**

C -0.161935 -0.408813 0.000012

N 1.080645 0.093898 -0.000027

H 1.237877 1.092568 0.000116

H 1.869027 -0.523041 0.000143

O -1.217188 0.152904 -0.000003

Vibrational frequency (cm^-1^)

*230.90*

*532.31*

*622.16*

*1096.28*

*1228.72*

*1616.48*

*1841.25*

*3522.02*

*3731.69*

**CONH_2__H_2__Int-2**

XYZ coordinates

C 0.272293 0.268010 -0.000045

O 1.466711 0.209037 -0.000087

H -2.460366 1.729444 -0.000083

N -0.640043 -0.712425 0.000106

H -0.359478 -1.683351 0.000207

H -1.615085 -0.483813 0.000120

H -3.163315 1.493576 -0.000025

Vibrational frequency (cm^-1^)

*48.34*

*79.30*

*149.20*

*155.01*

*251.53*

*322.81*

*532.34*

*630.27*

*1100.29*

*1230.58*

*1615.33*

*1840.46*

*3525.06*

*3727.25*

*4433.35*

**CONH_2__H_2__TS-2**

XYZ coordinates

C 0.160108 0.247780 -0.000028

O 1.229812 -0.299982 -0.000127

H 0.021929 1.523560 -0.000065

N -1.064853 -0.306722 0.000133

H -1.175205 -1.307577 0.000187

H -1.877427 0.282153 0.000201

H -0.249754 2.513774 -0.000074

Vibrational frequency (cm^-1^)

***1380.05i***

*265.36*

*288.07*

*351.29*

*549.45*

*639.77*

*1080.14*

*1136.00*

*1160.64*

*1306.38*

*1452.13*

*1624.24*

*1834.28*

*3576.80*

*3722.34*

**HCONH_2_ + H_Product_1 (Formamide)**

XYZ coordinates

C -0.152938 0.332723 0.004112

O -1.339907 0.076953 -0.000007

H 0.233783 1.363338 0.012090

N 0.838697 -0.593262 -0.000543

H 0.602181 -1.570320 -0.007563

H 1.803114 -0.318377 0.003808

H 3.015464 1.988192 -0.015912

Vibrational frequency (cm^-1^)

*36.66*

*37.44*

*130.40*

*210.16*

*566.41*

*641.13*

*1047.13*

*1055.05*

*1272.65*

*1419.81*

*1622.97*

*1774.45*

*2984.51*

*3596.62*

*3738.84*

**HCONH_TS-3**

XYZ coordinates

C -0.107960 0.235945 -0.035759

O -1.237432 -0.126935 0.011746

N 1.180090 -0.082603 -0.095865

H 1.443957 -0.925470 0.436789

H 0.789412 1.112143 0.369617

Vibrational frequency (cm^-1^)

1720.52*i*

464.18

533.71

744.25

1097.19

1170.74

1864.71

1994.11

3274.28

**HCONH_Int-3**

XYZ coordinates

C 0.0017553 0.0062994 -0.2949155

N -1.2267896 -0.5661081 0.1118937

H -1.9182231 -0.6112726 -0.6205979

H -1.1061200 -1.4688865 0.5494165

O 0.1109917 1.3584373 -0.0306118

N 1.1483780 -0.6988141 0.1314630

H 1.9509842 -0.0879250 0.1751236

H 1.3539505 -1.4977972 -0.4502975

H -0.6626829 1.6078647 0.4914300

Vibrational frequency (cm^-1^)

*9.35*

*45.95*

*125.94*

*203.27*

*248.56*

*334.94*

*515.91*

*937.65*

*1067.19*

*1226.85*

*1401.30*

*1675.38*

*3047.85*

*3450.91*

*4437.02*

**HCONH_H_2__Pre_Int-4**

XYZ coordinates

C 0.163577 -0.333990 -0.025784

O 1.371658 -0.200970 0.043300

H -0.321514 -1.308726 -0.150970

N -0.684431 0.752892 -0.107909

H -0.313592 1.515092 0.465023

H -3.452367 -1.216714 0.270318

H -3.076244 -0.648199 -0.020704

Vibrational frequency (cm^-1^)

*18.76*

*35.14*

*136.47*

*208.82*

*566.52*

*641.51*

*1047.05*

*1055.64*

*1272.82*

*1420.16*

*1622.60*

*1774.46*

*2984.99*

*3595.80*

*3738.19*

**HCONH_H_2__TS_4**

XYZ coordinates

C 0.233407 0.371739 -0.095203

O 1.315707 -0.132867 0.131228

H 0.106545 1.438630 -0.317644

N -0.917560 -0.375608 -0.226118

H -0.883700 -1.223029 0.340600

H -2.538275 0.810016 0.731976

H -2.006915 0.368365 0.250370

Vibrational frequency (cm^-1^)

*1167.25i*

*205.74*

*285.95*

*576.17*

*691.03*

*843.62*

*961.45*

*1041.78*

*1161.86*

*1233.72*

*1410.28*

*1707.06*

*2333.48*

*3031.57*

*3484.95*

**HCONH_2_ + H_Product-2 (Formamide)**

XYZ coordinates

C 0.159949 0.343826 0.003784

O 1.344715 0.078460 -0.013345

H -0.218015 1.377653 0.013095

N -0.839266 -0.573899 0.013740

H -0.610593 -1.552807 0.005863

H -3.231299 1.775903 -0.009830

H -1.801078 -0.290190 0.027038

**CONH_2__NH_3__Int-5**

XYZ coordinates

C -1.334033 -0.017932 -0.000045

N 2.058788 -0.059478 0.000075

H 2.657801 -0.102310 0.814792

H 1.486859 -0.898332 0.000082

O -0.951780 -1.158307 -0.000106

N -0.629176 1.115412 0.000043

H 0.394435 1.063152 0.000068

H -1.109735 1.993631 0.000067

H 2.657833 -0.102335 -0.814617

Vibrational frequency (cm^-1^)

95.38

104.62

154.08

203.84

220.50

373.59

483.21

575.26

794.54

1108.44

1129.17

1281.09

1607.53

1671.17

1681.70

1810.65

3289.27

3469.78

3589.55

3613.86

3715.64

**CONH_2__NH_3__TS-5**

XYZ coordinates

C 0.113925 0.172050 -0.111080

N -1.224707 -0.385306 -0.019488

H -1.358089 -1.266947 -0.503068

H -1.644290 -0.643663 1.166355

O 0.272884 1.373919 0.022869

N 1.093726 -0.779427 0.082328

H 2.028827 -0.407679 0.059681

H 0.989042 -1.688587 -0.332908

H -1.889077 0.301784 -0.362907

Vibrational frequency (cm^-1^)

1319.40*i*

203.95

376.94

433.48

489.91

505.89

590.38

774.86

907.33

955.07

1085.74

1172.95

1220.83

1328.63

1606.08

1640.09

1740.13

3474.02

3579.33

3588.73

**NH_2_CONH_2_ + H_Product_3 (Urea)**

XYZ coordinates

C 0.095020 -0.000019 0.007632

N -0.631613 1.158673 0.174042

H -1.631348 1.154880 0.084926

H -3.889878 0.015198 -0.121362

O 1.296484 0.004066 -0.199115

N -0.623528 -1.163656 0.175101

H -0.142167 -1.999261 -0.103849

H -1.623167 -1.167144 0.084922

H -0.156361 1.997467 -0.105774

Vibrational frequency (cm^-1^)

*19.19*

*52.48*

*147.28*

*167.24*

*376.43*

*399.84*

*480.19*

*548.98*

*571.05*

*772.88*

*961.59*

*1019.92*

*1175.00*

*1417.46*

*1632.51*

*1644.39*

*1770.96*

*3602.31*

*3610.95*

*3731.07*

**CONH_2__NH_3__ Protonated_TS-6**

XYZ coordinates

C 0.122263 0.080891 -0.349517

N -1.132011 -0.697925 0.068574

H -1.629825 -1.154963 -0.683807

H -0.982386 -1.331227 0.851115

O -0.257711 1.281269 0.048214

N 1.343364 -0.443405 0.130445

H 1.938833 0.312089 0.442751

H 1.830079 -1.017364 -0.541874

H -1.317272 0.506000 0.306799

Vibrational frequency (cm^-1^)

*1722.80i*

*276.49*

*328.97*

*411.55*

*508.95*

*677.21*

*731.10*

*805.50*

*987.73*

*1037.91*

*1078.54*

*1226.43*

*1394.54*

*1409.32*

*1571.15*

*1636.67*

*2063.74*

*3449.32*

*3533.92*

*3595.46*

*3627.75*

**NH_2_(C=O^+^)NH_2__NH_3__ Product-4 (Protonated Urea)**

XYZ coordinates

C 0.001755 0.006299 -0.294916

N -1.226790 -0.566108 0.111894

H -1.918223 -0.611273 -0.620598

H -1.106120 -1.468887 0.549417

O 0.110992 1.358437 -0.030612

N 1.148378 -0.698814 0.131463

H 1.950984 -0.087925 0.175124

H 1.353950 -1.497797 -0.450298

H -0.662683 1.607865 0.491430

Vibrational frequency (cm^-1^)

*130.30*

*263.04*

*417.44*

*453.48*

*476.63*

*560.16*

*703.31*

*789.54*

*901.29*

*1074.12*

*1112.53*

*1224.62*

*1347.97*

*1393.90*

*1637.80*

*1642.82*

*3538.19*

*3546.22*

*3633.60*

*3640.84*

*3748.55*

**CS**

XYZ coordinates

C 0.000000 0.000000 -1.118596

S 0.000000 0.000000 0.423494

Vibrational frequency (cm^-1^)

1276.01

**CS…NH_2__Pre-Reactant**

XYZ coordinates

C 2.228398 0.000006 -0.000011

N -2.731635 0.000001 0.000029

H -3.366068 -0.803880 0.000039

H -3.366058 0.803889 0.000039

S 0.687575 -0.000004 -0.000012

Vibrational frequency (cm^-1^)

9.54

11.22

76.72

99.30

142.86

1279.64

1543.79

3396.01

3489.68

**CS…NH_2__TS-1**

XYZ coordinates

C -0.294895 0.922048 0.056456

N 2.602198 -0.275164 -0.080917

H 2.337621 0.658212 -0.409065

H 2.950760 -0.097321 0.865817

S -1.212997 -0.312980 0.006216

Vibrational frequency (cm^-1^)

**58.30*i***

28.12

89.04

117.87

207.11

1283.87

1547.34

3394.15

3486.00

**CS…NH_2__Int-1**

XYZ coordinates

C 0.403799 -0.438489 0.000008

N 1.595561 0.122452 0.000057

H 1.718204 1.127715 0.000078

H 2.424498 -0.445366 0.000079

S -1.111856 0.069599 -0.000037

Vibrational frequency (cm^-1^)

*378.64*

*450.80*

*581.90*

*879.70*

*1158.90*

*1510.45*

*1641.84*

*3484.78*

*3663.49*

**CSNH_2__H_2__Int-2**

XYZ coordinates

C -0.289250 0.169161 -0.000004

H -2.526486 1.974347 -0.000045

N -1.302362 -0.670521 0.000148

H -1.170955 -1.674370 0.000251

H -2.245828 -0.322533 0.000166

H -3.266710 1.917767 0.000008

S 1.305980 0.062193 -0.000086

Vibrational frequency (cm^-1^)

70.00

89.25

177.04

215.81

365.61

386.33

467.94

590.70

880.33

1162.33

1513.60

1640.15

3487.49

3653.28

4414.68

**CSNH_2__H_2__TS-2**

XYZ coordinates

C -0.389486 0.315319 0.000020

H -0.789571 1.505879 -0.000016

N -1.481667 -0.441525 0.000179

H -1.418517 -1.446805 0.000237

H -2.390211 -0.007083 0.000244

H -1.380990 2.366992 -0.000012

S 1.180208 -0.077528 -0.000115

Vibrational frequency (cm^-1^)

**1405.83*i***

310.78

341.58

419.06

428.35

615.72

858.90

1097.55

1102.94

1320.32

1447.11

1516.09

1640.69

3550.98

3679.93

**HCSNH_2_ + H_Product_1 (Thioformamide)**

XYZ coordinates

C -0.407205 0.348383 0.000045

H -0.804459 1.361182 -0.000773

N -1.369263 -0.585262 -0.000281

H -1.113837 -1.558962 0.000317

H -2.341592 -0.332261 -0.001307

H -3.456623 1.920936 0.000201

S 1.209440 0.064720 0.001462

Vibrational frequency (cm^-1^)

41.02

49.62

151.43

357.57

434.22

628.49

886.58

966.86

1143.78

1314.52

1462.30

1641.22

3112.18

3569.21

3707.75

**HCSNH_TS-3**

XYZ coordinates

C 0.441261 0.267440 -0.052322

N 1.703057 -0.104674 -0.071254

H 1.949408 -1.012305 0.353004

H 1.356214 1.067445 0.368214

S -1.126392 -0.054866 0.006580

Vibrational frequency (cm^-1^)

**1745.77i**

382.08

488.37

678.54

843.30

1090.28

1546.61

2154.09

3231.49

**HCSNH_Int-3**

C -0.5795063021 0.4187862208 0.0010340339

N -1.5560619265 -0.3921549767 0.0002006841

H -2.4383686711 0.1141041013 -0.0002641869

H -0.6560707302 1.5109737009 0.0000273615

S 1.0938286299 -0.0893730463 -0.0005238926

**HCSNH_H_2__Pre_Int-4**

XYZ coordinates

C -0.4494787955 -0.2278868449 -0.0001107304

H -0.8137654823 -1.2533822697 -0.0007754945

N -1.2823630004 0.7336262222 -0.0021915287

H -0.8339751687 1.6499376287 -0.0013223179

H -3.6058551298 -1.5943289084 -0.0011140255

H -3.3119635538 -0.9123537106 -0.0006616388

S 1.2969851305 -0.0832631173 0.0039277359

Vibrational frequency (cm^-1^)

56.25

84.29

172.98

298.51

311.14

366.78

541.26

715.53

1063.12

1163.40

1373.81

1568.17

3110.35

3449.73

4410.43

**HCSNH_H_2__TS_4**

XYZ coordinates

C -0.3345757099 0.4738009021 -0.0851827155

H -0.5729337646 1.5301634097 -0.1826302844

N -1.3692381831 -0.3493210431 -0.1533922175

H -1.1889297869 -1.3068638437 0.1447443321

H -3.1998229964 0.4534158772 0.7648213475

H -2.4949847412 0.120394168 0.2955213005

S 1.2212911821 -0.0886714703 -0.0133027626

Vibrational frequency (cm^-1^)

***1686.19i***

*170.31*

*275.78*

*447.76*

*683.39*

*925.87*

*944.10*

*997.96*

*1117.49*

*1276.79*

*1347.92*

*1412.67*

*1842.37*

*3128.78*

**HCSNH_2_ + H_Product-2 (Thioformamide)**

XYZ coordinates

C 0.3599473297 0.3777247183 0.0009353351

H 0.7015371837 1.4105166828 0.0010406432

N 1.3700483528 -0.5033275278 -0.0001530266

H 1.166589849 -1.4892732525 -0.001518717

H 4.0946397903 1.3550955494 -0.0027661936

H 2.3279096739 -0.1993204245 -0.0013987751

S -1.2396021794 0.0083222544 0.0005587341

**CSNH_2__NH_3__Int-5**

XYZ coordinates

C -0.8789960697 0.7929436246 -0.0000215236

N 2.3182423032 -0.5631530323 0.0000980483

H 2.8822670679 -0.7672202863 0.8155456143

H 1.5396424313 -1.2150398347 -0.0000126333

N 0.2402841912 1.4757253561 0.0000392114

H 1.1498578211 0.9920463236 0.0000756335

H 0.2089489617 2.4806953699 0.0000531918

H 2.8824282435 -0.7671397169 -0.8152575333

S -1.2871289503 -0.7639148039 -0.000058009

Vibrational frequency (cm^-1^)

83.11

90.28

173.79

210.04

291.10

340.42

441.93

540.10

838.00

871.40

1112.21

1221.55

1517.61

1641.74

1674.12

1677.31

3190.01

3464.53

3583.97

3612.07

3638.84

**CSNH_2__NH_3__TS-5**

XYZ coordinates

C 0.262597 0.08324 -0.096192

N 1.080096 -1.058418 -0.040876

H 1.970624 -0.991921 -0.519944

H 1.535297 -1.447913 1.22607

N 0.958714 1.25123 0.077362

H 0.405176 2.088775 0.032366

H 1.902585 1.334551 -0.262558

H 0.562461 -1.879627 -0.330054

S -1.388962 -0.059562 0.010992

Vibrational frequency (cm^-1^)

**1258.22i**

213.47

353.15

386.71

427.75

464.21

497.50

732.83

810.48

933.44

1033.31

1113.53

1138.95

1338.69

1428.52

1611.18

1657.61

3495.97

3575.10

3600.04

**NH_2_CSNH_2_ + H_Product_3 (Thiourea)**

**XYZ coordinates**

C 0.2017981863 -0.0002351996 0.0001180534

N 0.9357604095 1.1446027417 -0.0488556947

H 1.8923585867 1.1430149816 0.2633432507

H 4.2111752708 0.0009866499 0.0076870621

N 0.9360306902 -1.145224883 0.0529936952

H 0.4210277007 -1.9995863197 -0.0610992106

H 1.8924746855 -1.1433662524 -0.2599642274

H 0.4208938 1.9996069323 0.0602477137

S -1.4635023298 -0.0004856507 -0.0036146423

Vibrational frequency (cm^-1^)

47.29

142.69

318.28

367.69

399.51

458.47

481.27

585.46

639.77

772.19

1070.51

1071.35

1416.45

1429.05

1634.70

1658.86

3574.88

3582.55

3711.36

**CSNH_2__NH_3__ Protonated_TS-6**

**XYZ coordinates**

C -0.393338 -0.072396 -0.306735

N -0.642689 1.350903 0.061556

H -1.046083 1.913473 -0.679782

H -1.198654 1.440300 0.912367

N -1.357408 -0.996332 0.130975

H -0.956478 -1.905397 0.308098

H -2.154226 -1.083698 -0.485596

H 0.675757 1.278144 0.229678

S 1.315024 -0.230653 0.012996

Vibrational frequency (cm^-1^)

**1571.65i**

228.33

266.49

344.28

440.25

543.24

690.92

727.56

908.13

1011.94

1065.35

1177.94

1232.11

1263.69

1584.72

1645.56

1727.94

3411.75

3520.92

3541.06

**NH_2_(C=S^+^)NH_2__NH_3__ Product-4 (Protonated Thiourea)**

XYZ coordinates

C -0.402446 -0.000010 0.225881

N -1.077623 -1.182183 -0.127246

H -0.596842 -2.004382 0.203796

H -2.047546 -1.200460 0.159642

N -1.077332 1.182313 -0.127189

H -0.597923 2.004365 0.206199

H -2.048097 1.199589 0.156835

H 1.457440 0.001213 -1.293259

S 1.342777 -0.000097 0.063817

Vibrational frequency (cm^-1^)

73.28

280.94

309.54

358.37

369.52

467.11

641.53

682.17

768.65

928.34

1055.86

1087.38

1343.02

1363.18

1626.87

1659.42

2488.00

3521.81

3522.34

3638.41

**
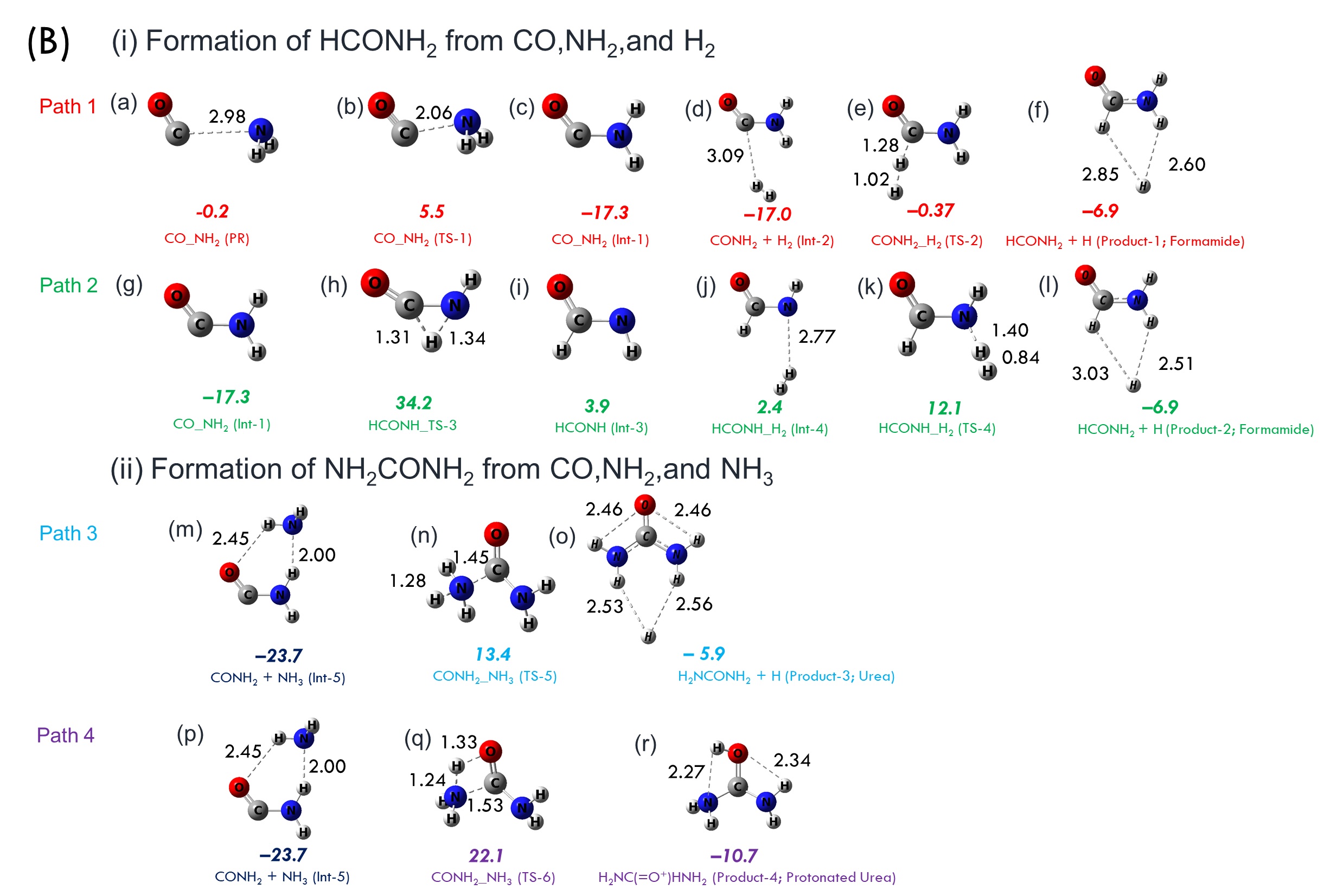
**

Figure SI_1 (i) Formation of HCONH_2_ from CO, NH_2_, and H_2_ (ii) Formation of NH_2_CONH_2_ from CO, NH_2_, and NH_3._ The zero-point corrected energy (kcal/mol) was calculated at CC-a/B2PD-a are relative to CO+NH_2_. All the bond distances are given in Å.


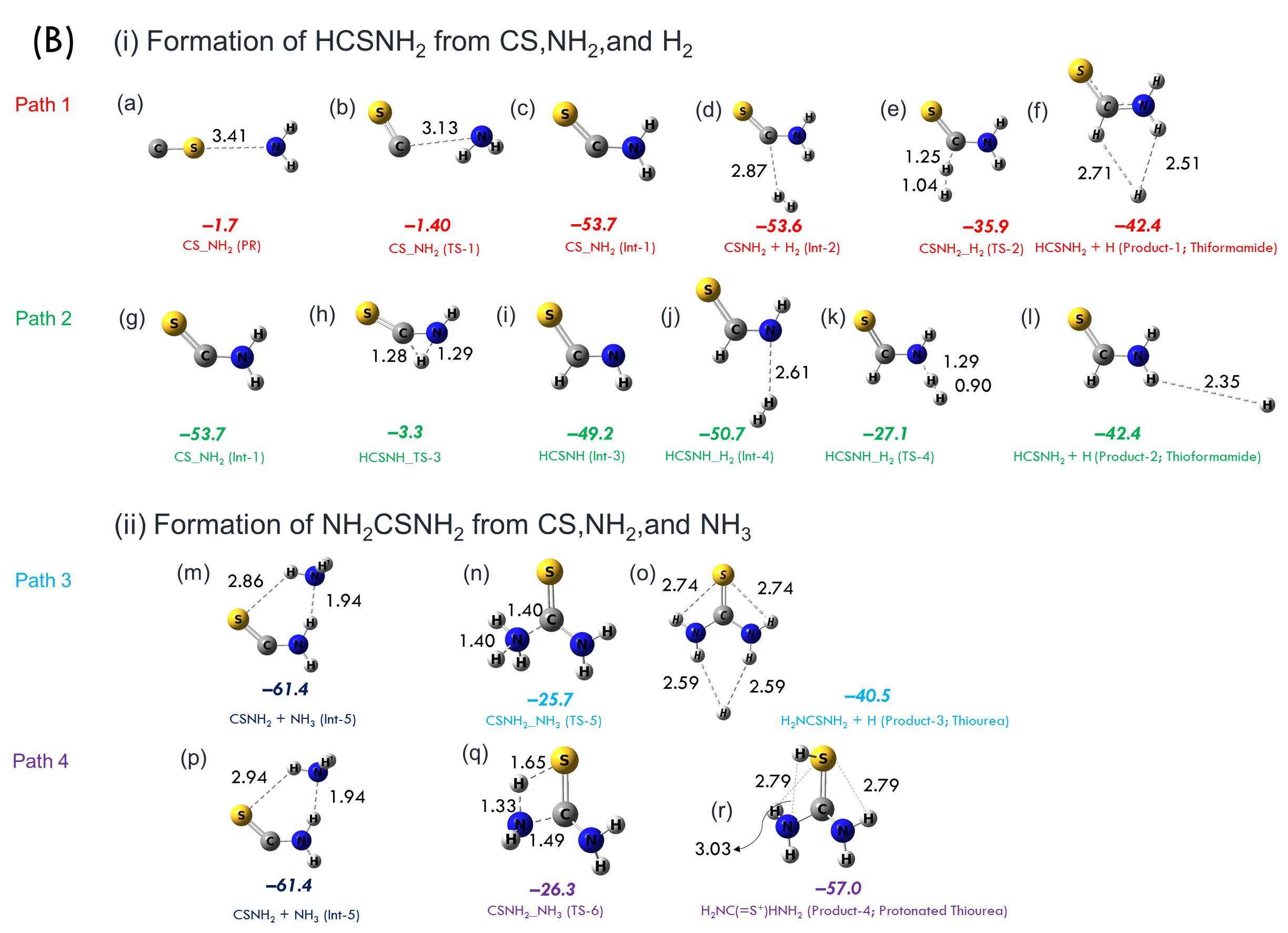


Figure SI_2. (i) Formation of HCSNH_2_ from CS, NH_2_, and H_2_ (ii) Formation of NH_2_CSNH_2_ from CS, NH_2_, and NH_3._ The zero-point corrected energy (kcal/mol) was calculated at CC-a/B2PD-a are relative to CS+NH_2_. All the bond distances are given in Å.

**Comparing the optimized geometries with their energy barriers with different DFT methods on representative TSs.**

Note: The same data presented in the form of energy values (Figure SI_1 to Figure SI_12) has been summarized in a Table 1 main text.

**
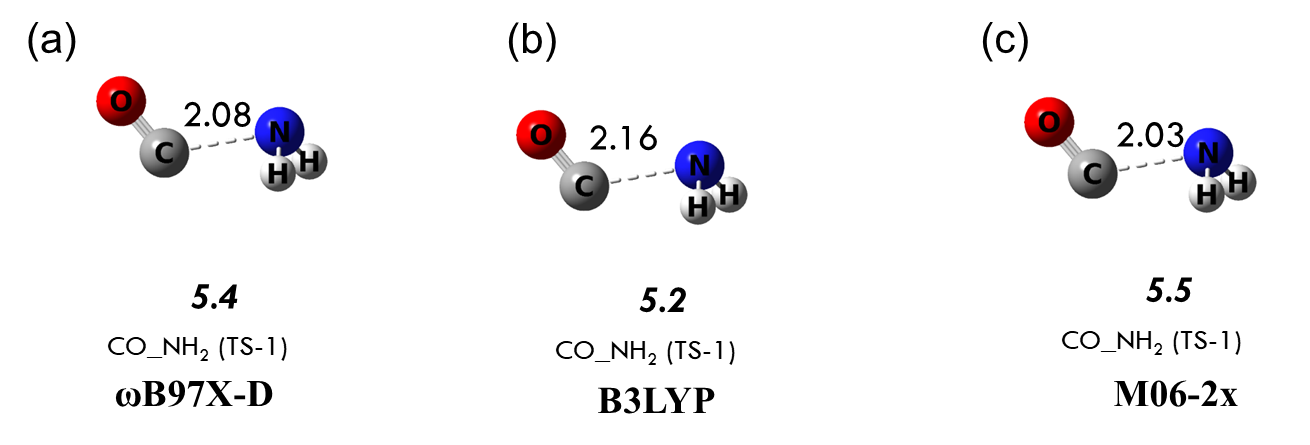
**

Figure SI_1: Optimized TSs of CO_NH_2__TS-1 (ωB97X-D). The geometry being calibrated with two different DFT (B3LYP, M06-2x) methods. Throughout, the aug-cc-pVTZ basis set was used for the optimization. All the resulting optimized geometries are similar. The UCCSD(T)(FC)/aug-cc-pVTZ level of theory is used for the single-point energy calculation. The electronic energy barriers (including zero-point corrections) are given below the structure and the values given in kcal/mol.

**
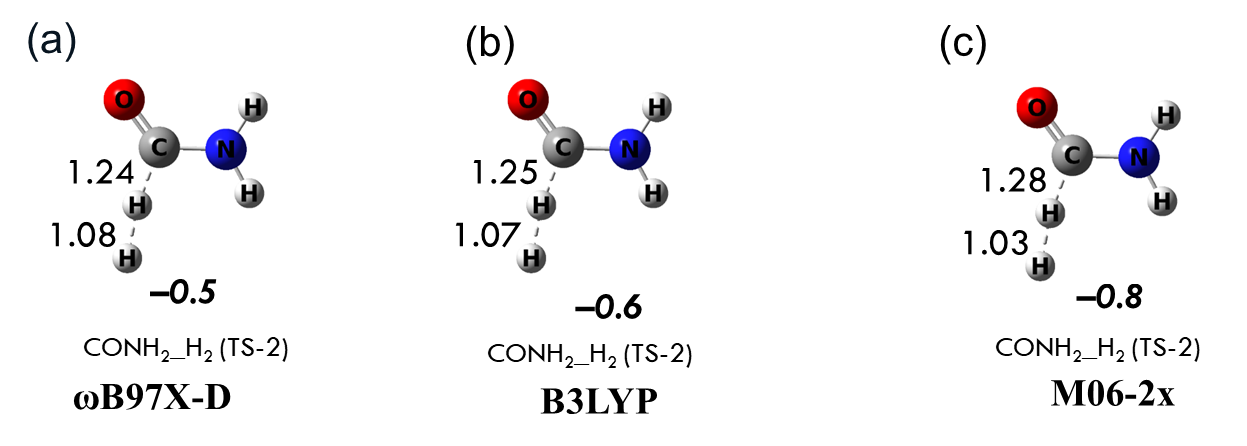
**

Figure SI_2: Optimized TSs of CONH_2__H_2__TS-2 (ωB97X-D). The geometry being calibrated with two different DFT (B3LYP, M06-2x) methods. Throughout, the aug-cc-pVTZ basis set was used for the optimization. All the resulting optimized geometries are similar. The UCCSD(T)(FC)/aug-cc-pVTZ level of theory is used for the single-point energy calculation. The electronic energy barriers (including zero-point corrections) are given below the structure and the values given in kcal/mol.

**
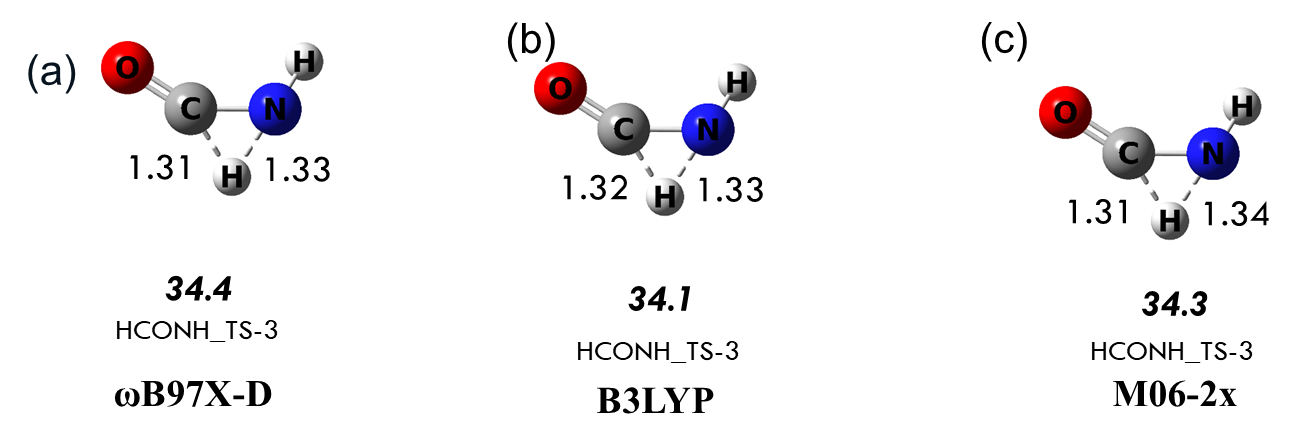
**

Figure SI_3: Optimized TSs of HCONH_TS-3 (ωB97X-D). The geometry being calibrated with two different DFT (B3LYP, M06-2x) methods. Throughout, the aug-cc-pVTZ basis set was used for the optimization. All the resulting optimized geometries are similar. The UCCSD(T)(FC)/aug-cc-pVTZ level of theory is used for the single-point energy calculation. The electronic energy barriers (including zero-point corrections) are given below the structure and the values given in kcal/mol.

**
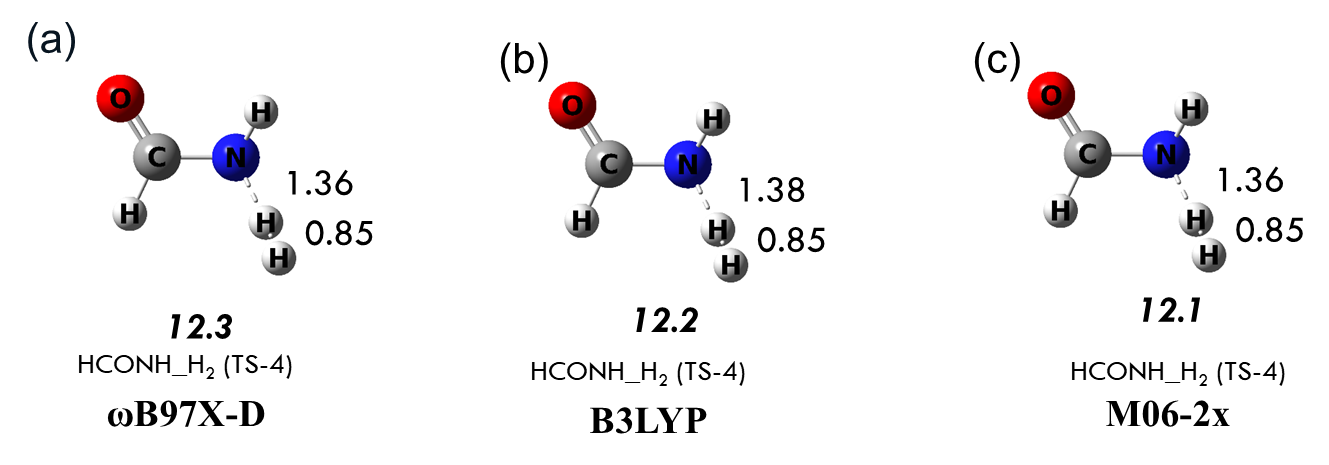
**

Figure SI_4: Optimized TSs of HCONH_H_2__TS-4 (ωB97X-D). The geometry being calibrated with two different DFT (B3LYP, M06-2x) methods. Throughout, the aug-cc-pVTZ basis set was used for the optimization. All the resulting optimized geometries are similar. The UCCSD(T)(FC)/aug-cc-pVTZ level of theory is used for the single-point energy calculation. The electronic energy barriers (including zero-point corrections) are given below the structure and the values given in kcal/mol.

**
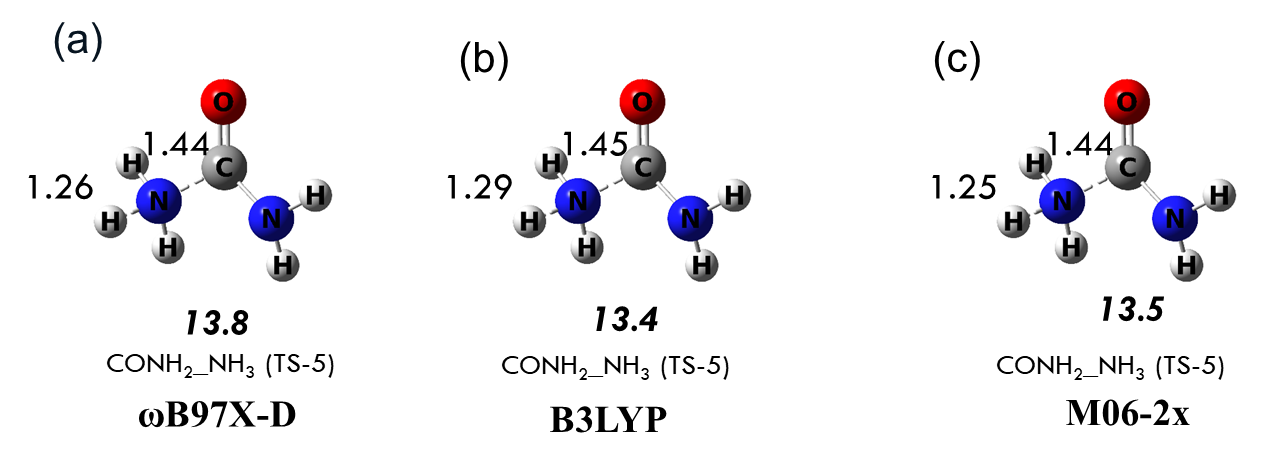
**

Figure SI_5: Optimized TSs of CONH_2__NH_3__TS-5 (ωB97X-D). The geometry being calibrated with two different DFT (B3LYP, M06-2x) methods. Throughout, the aug-cc-pVTZ basis set was used for the optimization. All the resulting optimized geometries are similar. The UCCSD(T)(FC)/aug-cc-pVTZ level of theory is used for the single-point energy calculation. The electronic energy barriers (including zero-point corrections) are given below the structure and the values given in kcal/mol.


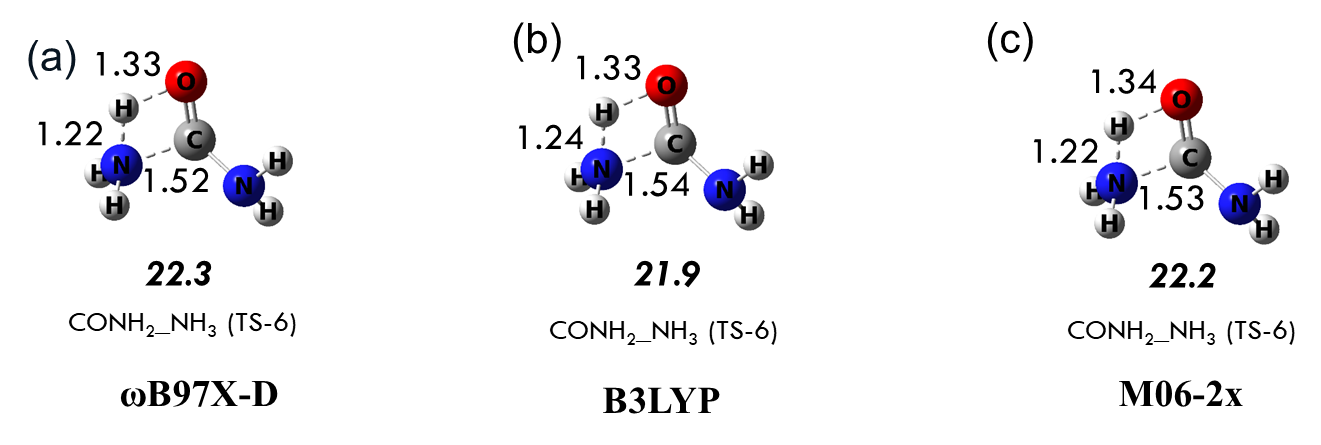


Figure SI_6: Optimized TSs of CONH_2__NH_3__TS-6 (ωB97X-D). The geometry being calibrated with two different DFT (B3LYP, M06-2x) methods. Throughout, the aug-cc-pVTZ basis set was used for the optimization. All the resulting optimized geometries are similar. The UCCSD(T)(FC)/aug-cc-pVTZ level of theory is used for the single-point energy calculation. The electronic energy barriers (including zero-point corrections) are given below the structure and the values given in kcal/mol.

**
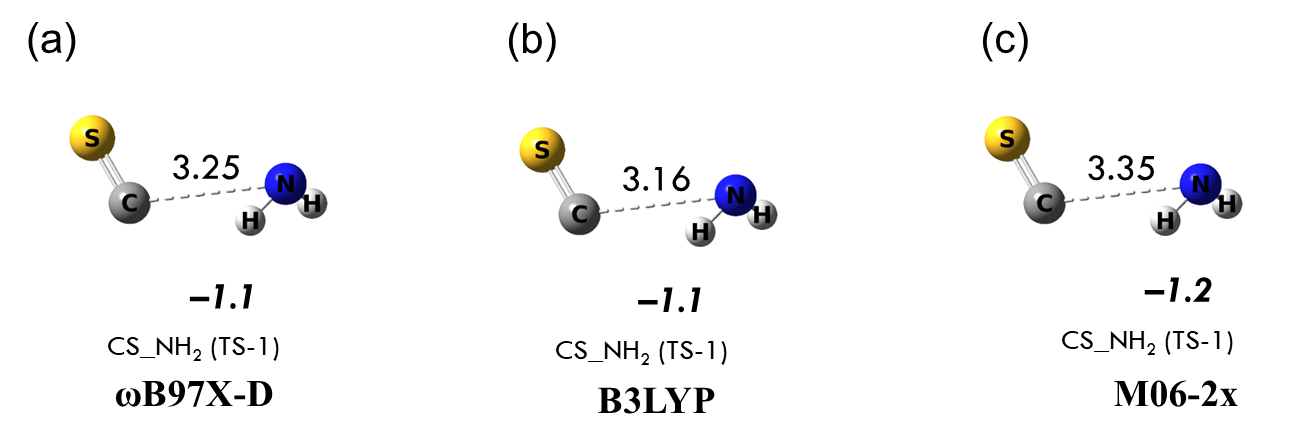
**

Figure SI_7: Optimized TSs of CS_NH_2__TS-1 (ωB97X-D). The geometry being calibrated with two different DFT (B3LYP, M06-2x) methods. Throughout, the aug-cc-pVTZ basis set was used for the optimization. All the resulting optimized geometries are similar. The UCCSD(T)(FC)/aug-cc-pVTZ level of theory is used for the single-point energy calculation. The electronic energy barriers (including zero-point corrections) are given below the structure and the values given in kcal/mol.

**
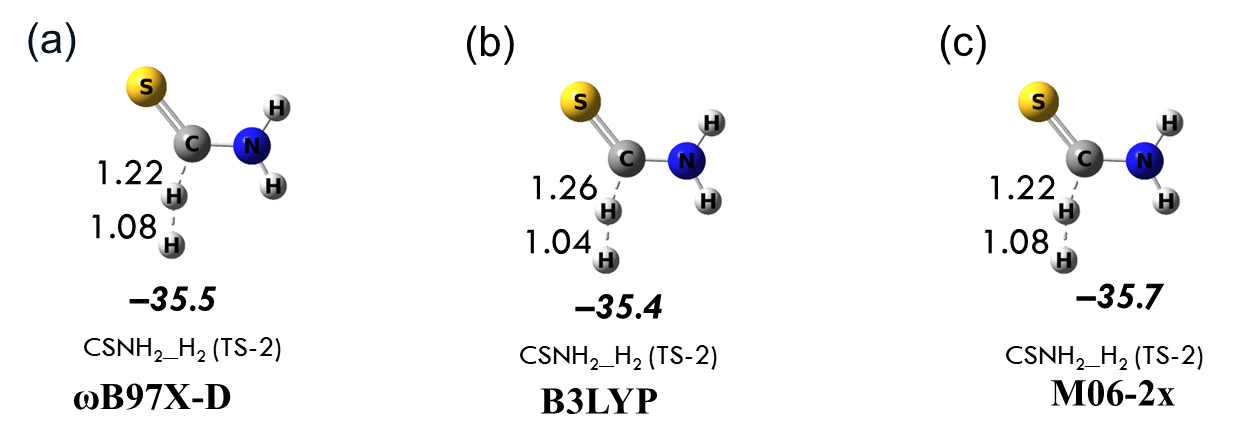
**

Figure SI_8: Optimized TSs of CSNH_2__H_2__TS-2 (ωB97X-D). The geometry being calibrated with two different DFT (B3LYP, M06-2x) methods. Throughout, the aug-cc-pVTZ basis set was used for the optimization. All the resulting optimized geometries are similar. The UCCSD(T)(FC)/aug-cc-pVTZ level of theory is used for the single-point energy calculation. The electronic energy barriers (including zero-point corrections) are given below the structure and the values given in kcal/mol.


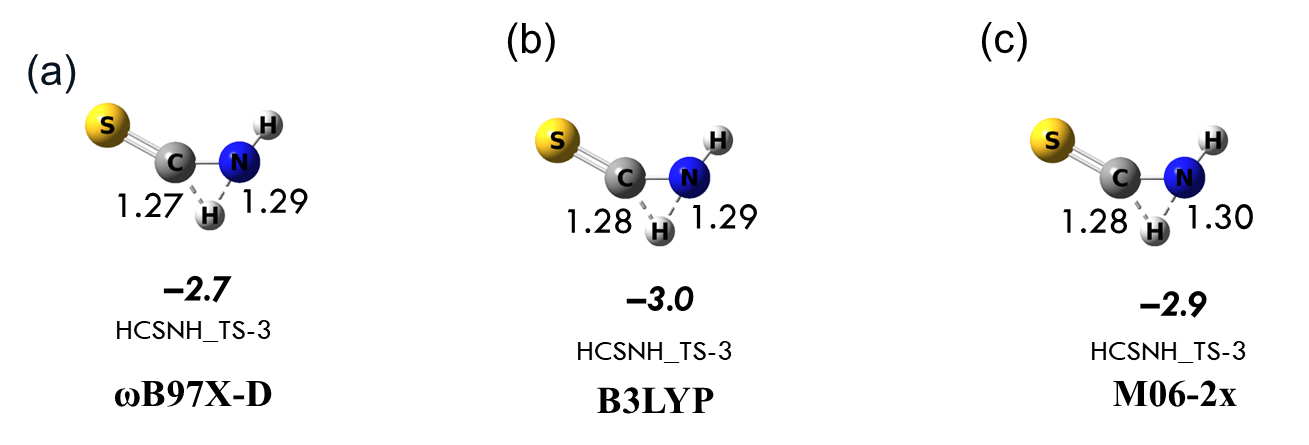
Figure SI_9: Optimized TSs of HCSNH_TS-3 (ωB97X-D). The geometry being calibrated with two different DFT (B3LYP, M06-2x) methods. Throughout, the aug-cc-pVTZ basis set was used for the optimization. All the resulting optimized geometries are similar. The UCCSD(T)(FC)/aug-cc-pVTZ level of theory is used for the single-point energy calculation. The electronic energy barriers (including zero-point corrections) are given below the structure and the values given in kcal/mol.

**
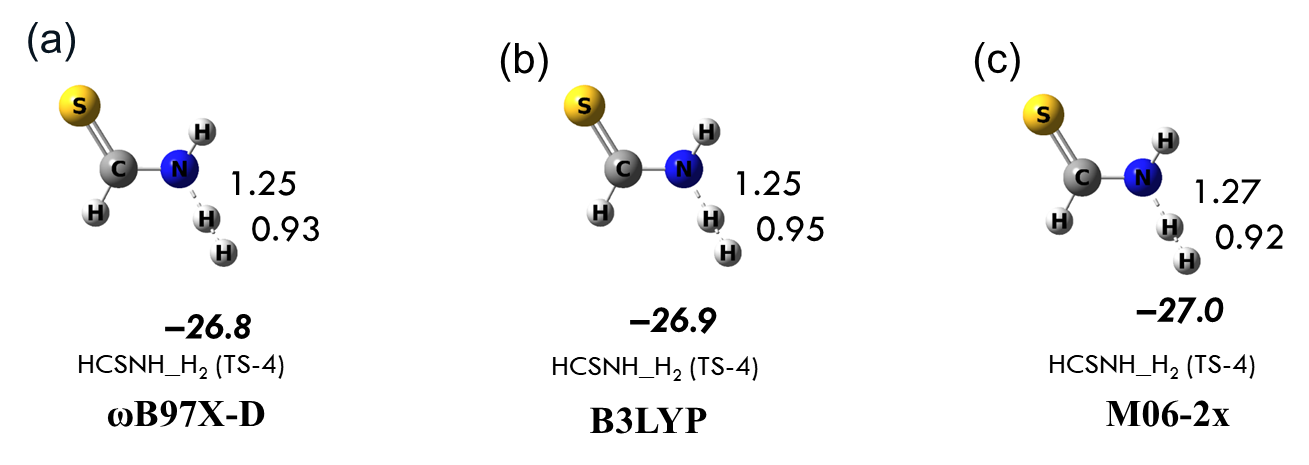
**

Figure SI_10: Optimized TSs of HCSNH_H_2__TS-4 (ωB97X-D). The geometry being calibrated with two different DFT (B3LYP, M06-2x) methods. Throughout, the aug-cc-pVTZ basis set was used for the optimization. All the resulting optimized geometries are similar. The UCCSD(T)(FC)/aug-cc-pVTZ level of theory is used for the single-point energy calculation. The electronic energy barriers (including zero-point corrections) are given below the structure and the values given in kcal/mol.

**
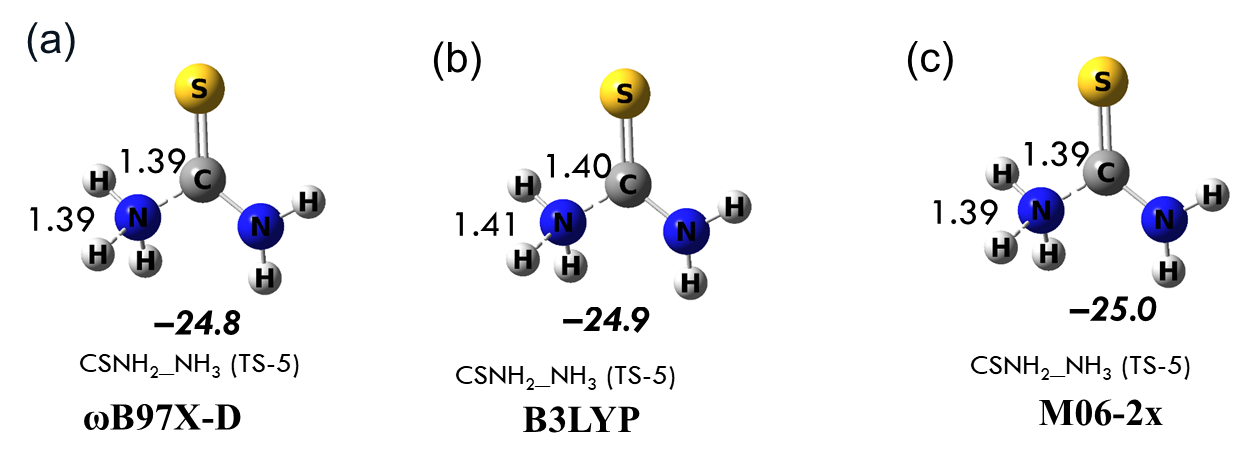
**

Figure SI_11: Optimized TSs of CSNH_2__NH_3__TS-5 (ωB97X-D). The geometry being calibrated with two different DFT (B3LYP, M06-2x) methods. Throughout, the aug-cc-pVTZ basis set was used for the optimization. All the resulting optimized geometries are similar. The UCCSD(T)(FC)/aug-cc-pVTZ level of theory is used for the single-point energy calculation. The electronic energy barriers (including zero-point corrections) are given below the structure and the values given in kcal/mol.

**
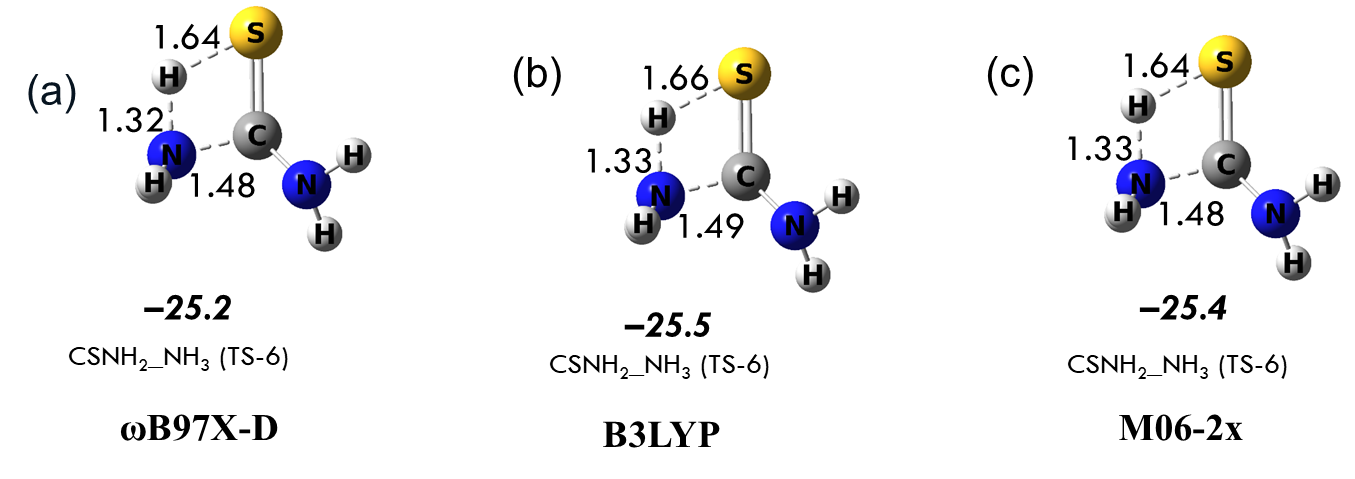
**

Figure SI_12: Optimized TSs of CSNH_2__NH_3__TS-6 (ωB97X-D). The geometry being calibrated with two different DFT (B3LYP, M06-2x) methods. Throughout, the aug-cc-pVTZ basis set was used for the optimization. All the resulting optimized geometries are similar. The UCCSD(T)(FC)/aug-cc-pVTZ level of theory is used for the single-point energy calculation. The electronic energy barriers (including zero-point corrections) are given below the structure and the values given in kcal/mol.

**Explaining how different sequences of two body reactions lead to the same highly stabilized pre-reaction complex.**

As part of this effort, we handle three entities: CS, NH_2_, and H_2_. However, it is evident that the occurrence of three-body collisions in low density gas-phase interstellar chemistry is quite improbable. Within this particular context, we assert unequivocally that we are not suggesting that *the pre-reactant complexes are forming through a three-body collision. Instead, they are generated through a sequential process that involves two bodies colliding.* The exact sequence is inconsequential because all of them lead to the same pre-reactant complex, and no barrier is encountered in the process. After the formation of the pre-reactant complex, the only process taking place is the molecular restructuring of this individual entity, leading to the transition state (TS), which subsequently produces the product complexes. Furthermore, irrespective of the specific sequence of two-body interactions that occur, the pre-reactant complex produced is consistently and considerably stabilized. The same level of theory used in the main-text is used here too. All energies reported are in kcal/mol.

1. The possible sequential two-body collisions for the formation of ***formamide***

*Possible collisions 1*

CO + NH_2_ → CO•••NH_2 ∆E = 0_

CO•••NH_2_ + H_2_ → HCONH_2_ + H _∆E = –14.8_

CO + NH_2_ + H_2_ → HCONH_2_ + H _∆E = –14.8_

*Possible Collision 2*

CO + H_2_ → CO•••H_2 ∆E = 0_

CO•••H_2_ + NH_2_ → HCONH_2_ + H _∆E = –14.9_

CO + H_2_ + NH_2_ → HCONH_2_ + H _∆E = –14.8_

The possible sequential two-body collisions for the formation of ***urea***

*Possible collisions 1*

CO + NH_2_ → CO•••NH_2 ∆E = 0_

CO•••NH_2_ + NH_3_ → NH_2_CONH_2_ + H _∆E = –15.1_

CO + NH_2_ + NH_3_ → NH_2_CONH_2_ + H _∆E = –15.1_

*Possible collisions 2*

CO + NH_3_ → CO•••NH_3 ∆E = –0.1_

CO•••NH_3_ + NH_2_ → NH_2_CONH_2_ + H _∆E = –14.9_

CO + NH_3_ + NH_2_ → NH_2_CONH_2_ + H _∆E = –15.1_

1. The possible sequential two-body collisions for the formation of ***thioformamide***

*Possible collisions 1*

CS + NH_2_ → CS•••NH_2 ∆E = –0.9_

CS•••NH_2_ + H_2_ → HCSNH_2_ + H _∆E = –50.5_

CS + NH_2_ + H_2_ → HCSNH_2_ + H _∆E = –51.4_

*Possible collisions 2*

CS + H_2_ → CS•••H_2 ∆E = 0.2_

CS•••H_2_ + NH_2_ → HCSNH_2_ + H _∆E = –51.6_

CS + H_2_ + NH_2_ → HCSNH_2_ + H _∆E = –51.4_

The possible sequential two-body collisions for the formation of ***thiourea***

*Possible collisions 1*

CS + NH_2_ → CS•••NH_2 ∆E = –0.9_

CS•••NH_2_ + NH_3_ → NH_2_CSNH_2_ + H _∆E = –49.9_

CS + NH_2_ + NH_3_ → NH_2_CSNH_2_ + H _∆E = –50.8_

*Possible collisions 2*

CS + NH_3_ → CS•••NH_3 ∆E = –1.2_

CS•••NH_3_ + NH_2_ → NH_2_CSNH_2_ + H _∆E = –49.6_

CS + NH_3_ + NH_2_ → NH_2_CSNH_2_ + H _∆E = –50.8_

All energies reported are in kcal/mol.

**The energy difference between products and reactants of** **Formamide, Urea, Thioformamide, and Thiourea.**

The B2PLYP-D/aug-cc-pVTZ level of theory used, the reactions are exothermic as stated in the main-text.

Table SI_1: The Reaction of CO + NH_2_ + H_2_ → HCONH_2_ + H^.^

| **Reactants** | **Products** | **Energy(ΔE)** |
| --- | --- | --- |
| CO + NH_2_ + H_2_ | HCONH_2_ + H^.^ | ^–14.9^ |

Table SI_2: The Reaction of CO + NH_2_ + NH_3_ → H_2_NCONH_2_ + H^.^

| **Reactants** | **Products** | **Energy(ΔE)** |
| --- | --- | --- |
| CO + NH_2_ + NH_3_ | H_2_NCONH_2_ + H^.^ | ^–15.2^ |

Table SI_3: The Reaction of CS + NH_2_ + H_2_ → HCSNH_2_ + H^.^

| **Reactants** | **Products** | **Energy(ΔE)** |
| --- | --- | --- |
| CS + NH_2_ + H_2_ | HCSNH_2_ + H^.^ | ^–42.4^ |

Table SI_4: The Reaction of CS + NH_2_ + H_2_ → H_2_NCSNH_2_ + H^.^

| **Reactants** | **Products** | **Energy(ΔE)** |
| --- | --- | --- |
| CS + NH_2_ + NH_3_ | H_2_NCSNH_2_ + H^.^ | ^–40.4^ |

All energies reported are in kcal/mol.

**Table SI_5. Imaginary frequencies the Formamide, Urea, Thioformamide, and Thiourea TSs**

| **Formamide and Urea TSs** | **ωB97X-D** | **B3LYP** | **M06-2x** | **B2LYP-D** |
| --- | --- | --- | --- | --- |
| CO_NH_2__TS-1 | 294.52i | 207.75i | 368.34i | 301.62i |
| CONH_2__H_2__TS-2 | 1163.84i | 1039.28i | 1795.40i | 1380.04i |
| HCONH_TS-3 | 1776.67i | 1752.37i | 1684.37i | 1720.51i |
| HCONH_H_2__TS-4 | 1242.49i | 1046.23i | 1258.05i | 1167.25i |
| CONH_2__NH_3__TS-5 | 1250.38i | 1159.16i | 1211.14i | 1319.39i |
| CONH_2__NH_3__TS-6 | 1722.36i | 1726.91i | 1564.39i | 1722.80i |
|  |  |  |  |  |
| **Thioformamide and Thiourea TSs** | **ωB97X-D** | **B3LYP** | **M06-2x** | **B2LYP-D** |
| CS_NH_2__TS-1 | 79.36i | 82.17i | 87.93i | 58.30i |
| CSNH_2__H_2__TS-2 | 1255.31i | 1042.27i | 1815.66i | 1405.82i |
| HCSNH_TS-3 | 1821.28i | 1815.55i | 1727.08i | 1745.77i |
| HCSNH_H_2__TS-4 | 1812.23i | 1622.71i | 2035.84i | 1686.18i |
| CSNH_2__NH_3__TS-5 | 1355.51i | 1116.89i | 1325.50i | 1258.21i |
| CSNH_2__NH_3__TS-6 | 1526.38i | 1549.61i | 1535.54i | 1571.65i |

All energies reported are in kcal/mol.

**Table_SI_6. The electronic energies for all investigated compounds (in Hartrees)**

|  | Zero-point correction  **B2PLYP-D** | UCCSD(T) | UCCSD(T)+ Zero-point correction |
| --- | --- | --- | --- |
| CO_NH_2_(Pre-Reactant) | 0.02531 | -168.963494 | -168.9381883 |
| CO_NH_2__TS-1 | 0.02794 | -168.957003 | -168.9290589 |
| CO_NH_2__Int-1 | 0.03286 | -168.998398 | -168.9655434 |
| CONH_2_+H_2__Int-2 | 0.04475 | -170.172362 | -170.1276157 |
| CONH_2__H_2__TS-2 | 0.04326 | -170.144232 | -170.1009771 |
| HCONH_2_+H_Product-1 (Formamide) | 0.04587 | -170.157253 | -170.1113844 |
| HCONH_TS-3 | 0.02539 | -168.908775 | -168.8833887 |
| HCONH_Int-3 | 0.03025 | -168.961907 | -168.9316601 |
| HCONH_H_2__Int-4 | 0.04267 | -170.139161 | -170.0964952 |
| HCONH_H_2__TS-4 | 0.04321 | -170.124247 | -170.0810331 |
| HCONH_2_+H_Product-2 (Formamide) | 0.04583 | -170.157232 | -170.1113981 |
| CONH_2_+NH_3__Int-5 | 0.06693 | -225.429497 | -225.3625648 |
| CONH_2__NH_3__TS-5 | 0.06341 | -225.456933 | -225.3935188 |
| H_2_NCONH_2_+H_Product-3 (Urea) | 0.06915 | -225.417928 | -225.348781 |
| CONH_2__NH_3__TS-6 | 0.07344 | -225.474607 | -225.4011672 |
| H_2_NCONH_2_+H_Product-4(Protonated Urea) | 0.02531 | -168.963494 | -168.9381883 |
|  |  |  |  |
|  |  |  |  |
|  |  |  |  |
|  | Zero-point correction  **B2PLYP-D** | UCCSD(T) | UCCSD(T)+ Zero-point correction |
| CS_NH_2_(Pre-Reactant) | 0.022893 | -491.5025222 | -491.4796292 |
| CS_NH_2__TS-1 | 0.023131 | -491.5023692 | -491.4792382 |
| CS_NH_2__Int-1 | 0.031326 | -491.5939881 | -491.5626621 |
| CSNH_2_+H_2__Int-2 | 0.043546 | -492.7684493 | -492.7249033 |
| CSNH_2__H_2__TS-2 | 0.041759 | -492.7384292 | -492.6966702 |
| HCSNH_2_+H_Product-1 (Thioformamide) | 0.044349 | -492.7513523 | -492.7070033 |
| HCSNH_TS-3 | 0.023727 | -491.5060492 | -491.4823222 |
| HCSNH_Int-3 | 0.030489 | -491.5859644 | -491.5554754 |
| HCSNH_H2_Int-4 | 0.042569 | -492.7628073 | -492.7202383 |
| HCSNH_H2_TS-4 | 0.041129 | -492.7235988 | -492.6824698 |
| HCSNH_2_+H_Product-2 (Thioformamide) | 0.044239 | -492.7512874 | -492.7070484 |
| CSNH_2_+NH_3__Int-5 | 0.064958 | -548.0290529 | -547.9640949 |
| CSNH_2__NH_3__TS-5 | 0.061682 | -548.0493163 | -547.9876343 |
| H_2_NCSNH_2_+H_Product-3 (Thiourea) | 0.065965 | -548.0310226 | -547.9650576 |
| CSNH_2__NH_3__TS-6 | 0.067946 | -548.0819001 | -548.0139541 |
| H_2_NCSNH_2_+H_Product-4(Protonated Thiourea) | 0.022893 | -491.5025222 | -491.4796292 |

**Table_SI_7**: The Equilibrium constant and rate constants (in cm^3^ molecule^-1^ s^-1^) for CS+NH_2_→ CS…NH_2_

| Temp | **Ke** | **micro-k1** |
| --- | --- | --- |
| 10 | 2.21E+14 | 7.59E-09 |
| 15 | 1.09E+02 | 3.84E-09 |
| 20 | 7.14E-05 | 2.24E-09 |
| 25 | 1.35E-08 | 1.42E-09 |
| 30 | 4.39E-11 | 9.55E-10 |
| 35 | 7.35E-13 | 6.66E-10 |
| 40 | 3.44E-14 | 4.79E-10 |
| 45 | 3.20E-15 | 3.53E-10 |
| 50 | 4.84E-16 | 2.65E-10 |
| 55 | 1.04E-16 | 2.02E-10 |
| 60 | 2.91E-17 | 1.57E-10 |
| 65 | 9.99E-18 | 1.23E-10 |
| 70 | 4.03E-18 | 9.76E-11 |
| 75 | 1.85E-18 | 7.84E-11 |
| 80 | 9.40E-19 | 6.35E-11 |
| 85 | 5.21E-19 | 5.20E-11 |
| 90 | 3.10E-19 | 4.29E-11 |
| 100 | 1.96E-19 | 2.98E-11 |


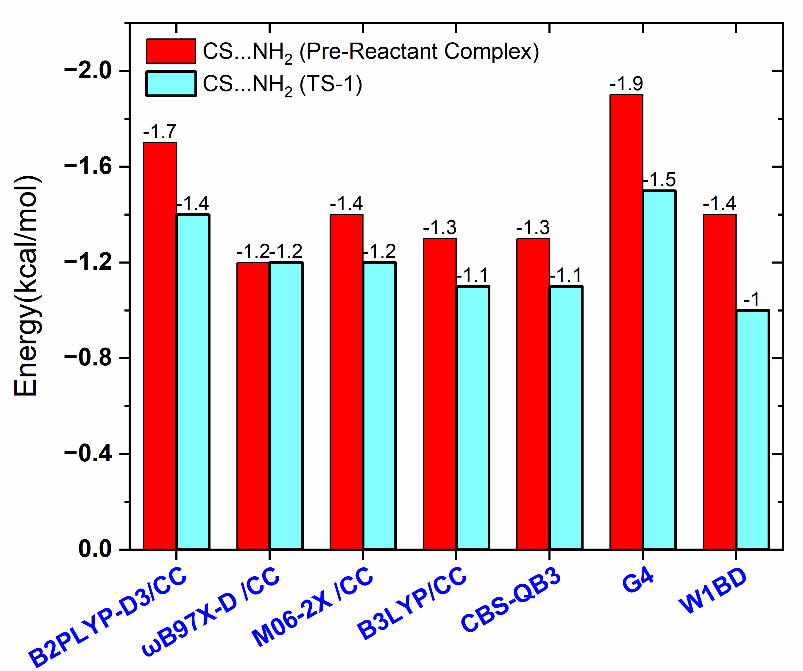


Figure SI_13: Zero-point corrected energies of pre-reactive complex and TS-1 in the case of CS+NH_2_ reaction at different levels of theories.
